# Toward Artificial Mussel‐Glue Proteins: Differentiating Sequence Modules for Adhesion and Switchable Cohesion

**DOI:** 10.1002/anie.202008515

**Published:** 2020-08-19

**Authors:** Sandra Arias, Shahrouz Amini, Justus Horsch, Matthias Pretzler, Annette Rompel, Inga Melnyk, Dmitrii Sychev, Andreas Fery, Hans G. Börner

**Affiliations:** ^1^ Laboratory for Organic Synthesis of Functional Systems Department of Chemistry Humboldt-Universität zu Berlin Brook-Taylor-Straße 2 12489 Berlin Germany; ^2^ Max Planck Institute of Colloids and Interfaces Department of Biomaterials 14424 Potsdam Germany; ^3^ Universität Wien Fakultät für Chemie Institut für Biophysikalische Chemie Althanstraße 14 1090 Wien Austria; ^4^ Leibniz-Institut für Polymerforschung Dresden e.V. Institute of Physical Chemistry and Polymer Physics Hohe Straße 6 01069 Dresden Germany; ^5^ Technische Universität Dresden Chair of Physical Chemistry of Polymeric Materials Hohe Straße 6 01069 Dresden Germany

**Keywords:** adhesion, cohesion control, enzyme-induced polymerization, mussel glue, synthetic proteins

## Abstract

Artificial mussel‐glue proteins with pH‐triggered cohesion control were synthesized by extending the tyrosinase activated polymerization of peptides to sequences with specific modules for cohesion control. The high propensity of these sequence sections to adopt β‐sheets is suppressed by switch defects. This allows enzymatic activation and polymerization to proceed undisturbed. The β‐sheet formation is regained after polymerization by changing the pH from 5.5 to 6.8, thereby triggering O→N acyl transfer rearrangements that activate the cohesion mechanism. The resulting artificial mussel glue proteins exhibit rapid adsorption on alumina surfaces. The coatings resist harsh hypersaline conditions, and reach remarkable adhesive energies of 2.64 mJ m^−2^ on silica at pH 6.8. In in situ switch experiments, the minor pH change increases the adhesive properties of a coating by 300 % and nanoindentation confirms the cohesion mechanism to improve bulk stiffness by around 200 %.

## Graphical abstract

Artificial protein analogues of adhesive mussel proteins were prepared by enzyme‐induced peptide polymerization, integrating sequence modules for adhesion and activable cohesion control. The polymers are generated by the formation of cysteinyldopa linkages and combine potent interaction functionalities with a pH‐triggered cohesion‐control mechanism, thus enabling β‐sheet formation and getting closer to mimicking mussel foot protein.

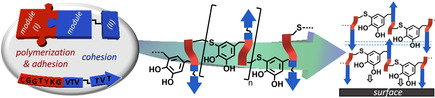

Water‐based wet adhesives, that tolerate hostile conditions are of high interest as they promise resistant under water glues, surgical sealants as well as biofriendly dyes, anti‐fouling or anti‐corrosive coatings.^1^, ^2^ One of the most prominent bioadhesion systems originates from marine mussels.^3^ Progress in understanding the sequence‐structure‐function relationships of mussel foot proteins (mfps) revealed a concerted “reactive molding” process of different mfps devoted to specific tasks by constituting byssus, adhesive plaque and material‐specific adhesive interfaces.^4^, ^5^

Within the last decades, several mfps were recombinantly expressed and structural control was achieved by fusing mfps with Amyloid segments.^6^ Nonetheless the complexity in structure and function limits ease of rational adaptation of mfps to tailor properties. Mussel‐glue inspired polymers are more straightforward to synthesize, due to a reduced complexity compared to mfps.^7^, ^8^, ^9^ They present l‐3,4‐dihydroxyphenylalanine (Dopa) or catechol derivatives to constitute both adhesion to various surfaces^10^, ^11^ and cohesion by either covalent, for example, di/oligoDopa formation^12^ or noncovalent interactions, for example, Fe^3+^ complexation.^13^ The rich set of mussel‐glue inspired polymers enables exciting applications.^14^ However, if compared to mfps many opportunities can still be explored.

Waite et al. demanded to proceed beyond exclusively Dopa‐carrying synthetic homologues, as the sequence environment is of relevance in the biological blueprint.^11^ Early work was pioneered by Messersmith et al. accompanying Dopa with Lys to improve adhesion of mussel‐glue inspired polymers.^2^ NMR data of a mussel‐inspired 12*mer* peptide^9^ underlined the importance of the sequence by giving molecular insights into peptide adsorption to Al_2_O_3_ and proving the sequence environment of Dopa to modulate adsorption and structural properties.^15^

Recently, a tyrosinase activated polymerization of peptides, containing Cys and Tyr residues, was reported to broaden sequence complexity in mussel‐glue inspired polymers.^16^, ^17^ The Tyr residues were enzymatically oxidized to Dopa‐quinones, to which thiols of Cys could link by an intermolecular Michael‐addition. The resulting polymers adsorb strongly to various surfaces with high adhesion energies.^16^ NaIO_4_ allows chemical activation of peptides with Dopa instead of Tyr. This enables the polymerization of Dopa‐Lys‐Cys minimal‐sequences independent of a substrate aptitude^17^ or the crosslinking into hydrogels.^8^

While primarily adhesion properties were in the focus of artificial mussel‐glue proteins, other features of the mussel glue apparatus appear of key interest, too. For instance, mfps of the byssus thread exhibit β‐sheets connected by unstructured regions to adjust filament mechanics.^5^ Thus equipping artificial mussel‐glue proteins with controlled cohesion mechanisms is of interest.

Here we expand the concept of artificial mussel‐glue proteins by integrating a sequence module for cohesion control. Tyrosinase activated polymerization of functionally differentiated peptides was applied. Those unimers fuse the Tyr/Cys‐bearing segment required for enzymatic polymerization,^16^ with a (Val‐Thr)_*n*_ domain to improve cohesion by β‐sheet formation (Figure 1). An interference with the tyrosinase activation and polymerization steps was prevented by embedding depsi‐switch defects^18^ into the (VT)_*n*_ domain to reduce β‐sheet propensity but permit regaining β‐sheet formation by minor pH changes.

**Figure 1:**
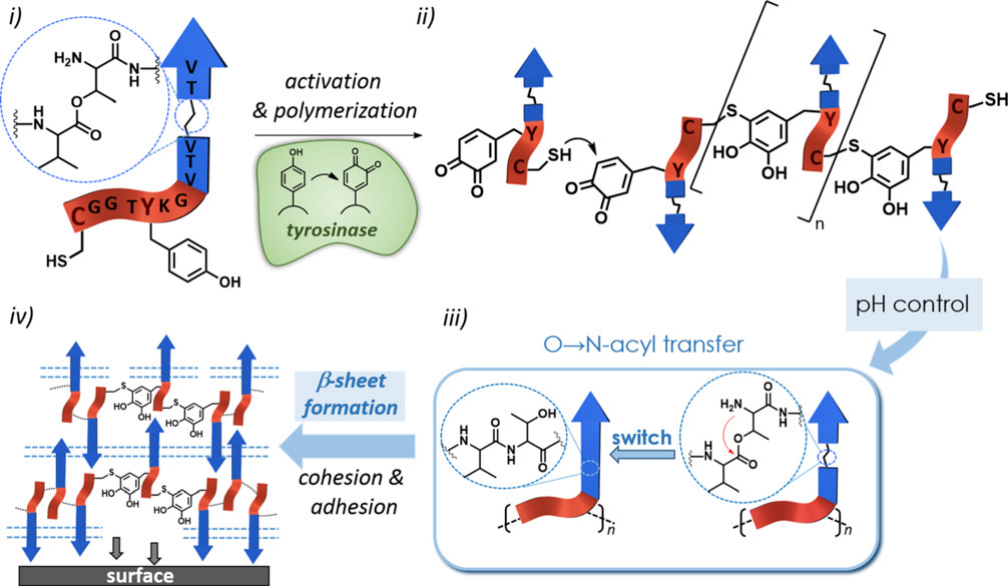
Illustration of the mussel‐inspired polymerization of disturbed [C/Y]‐(VT)_*n*_^switch^ unimers (i) that combine a polymerization module (red) with a (VT)_*n*_^switch^ cohesion control module (blue). The enzymatic oxidation of tyrosine residues to Dopa‐quinones induces peptide polymerization in which cysteinyldopa connectivities are formed (ii). The suppressed β‐sheet formation is regained by pH changes (iii), leading to mfp analogues with strong adhesion and cohesion properties (iv).

The C‐terminal domain TYK of a recently polymerized mfp‐1 consensus sequence AKPSYPPT**Y**K allowed for fast and complete oxidation by tyrosinase. The N‐terminal flank was used to position Cys via a GG‐spacer, yielding the CGGT**Y**K ([C/Y]) module, which is required for enzymatic activation and polymerization. This module was complemented C‐terminally via a G‐spacer with a cohesion module, composed of a (VT)_*n*_ segment, having high β‐sheet propensities. A set of CGGT**Y**KG(VT)_*n*_ unimers with *n*=1–3 was synthesized (S.I.).

The unimer activation that induces polymerization was carried out with a recombinant tyrosinase (*Agaricus bisporus* polyphenol oxidase isoform 4, *Ab*PPO4).^19^ Depending on the length of the cohesion module, strong interferences with the enzymatic activation and polymerization process were evidenced (S.I. Figure S10). While [C/Y]‐(VT)_1_ polymerized cleanly, [C/Y]‐(VT)_2_ forms gels during polymerization, and [C/Y]‐(VT)_3_ directly shows gel formation prior to activation. These results were expected, due to the high β‐sheet tendency of (VT) domains,^20^ making handling of unimers and polymers difficult (S.I. Figure S10b).

To suppress the aggregation of (VT)_*n*_ domains, unimers containing depsipeptide connectivities in the VT segments were synthesized. Those structural defects were shown to improve the synthesis of β‐Amyloid_42_ 21 and offered tools to regulate the aggregation tendency of β‐sheet builders^18^ or collagen mimetic peptides.^22^ The depsipeptides are referred to as “switch”^21^ peptides, having ester connectivities between for example, a Thr(*n*) β‐OH and Val(*n*+1) α‐COOH that leads to (VT)^Ψ^ O‐acyl isomers. The native [C/Y]‐(VT)_*n*_ unimers were accompanied with a set of switch‐peptides. Besides, VT‐all‐switch peptides like [C/Y]‐(VT)_1_^Ψ9^, [C/Y]‐(VT)_2_^Ψ9,11^ and [C/Y]‐(VT)_3_^Ψ9,11,13^, the higher homologs [C/Y]‐(VT)_4_^Ψ9,13^ and [C/Y]‐(VT)_5_^Ψ11,15^ included two switches. The depsi‐structures constitute reversible defects that can be converted back to the native peptide connectivity to regain β‐sheet propensities as observed by circular dichroism spectroscopy (S.I. Figure S14). The O→N‐acyl transfer is controlled by minor pH changes. While a shift in pH from pH 5.5→pH 6.5 gives slow switching kinetics,^18^ the switch at pH 7.4 was reported to proceed with a half‐life time of *τ*_1/2_≈1 minutes.^21^ Infrared (IR) spectroscopy confirms the effective switching of the unimers by proving the absence of depsi‐ester bands at pH 6.8 (S.I. Figure S9).

*Ab*PPO4 shows the highest activity at pH 6–7,^23^ where the O→N‐acyl rearrangement is triggered. However, UV/vis spectroscopy confirmed rapid enzymatic activation of all [C/Y]‐(VT)_*n*_^Ψ^ unimers at pH 5.5, at which switch‐segments proved to be stable (S.I. Figure S12). Only minor differences in the initial activation rates were found between [C/Y]‐(VT)_1_^Ψ9^ and [C/Y]‐(VT)_5_^Ψ11,15^. The positive charges affect tyrosinase activity marginally as unimers with three switches were activated with slightly reduced rates (S.I. Figure S12). Interestingly, no differences in activation kinetics of [C/Y]‐(VT)_1_^Ψ9^ and [C/Y]‐(VT)_1_ were found. This suggests that neither the structural defects, nor the positive charge of one depsi‐segment interfere with unimer activation.

The enzymatic activation of [C/Y]‐(VT)^Ψ^ yielded in all cases poly([C/Y]‐(VT)_*n*_^Ψ^) as shown by SDS‐PAGE and GPC analysis (S.I. Figures S11&S13, Table S2). Considering that tyrosinase oxidation of phenols proceeds in the catalytic cycle all the way to *o*‐quinones,^24^ which react fast with thiol‐nucleophiles,^25^ the UV/vis monitoring of tyrosinase activation at 293 nm suggested the formation of cysteinyldopa to be accomplished within 10–20 minutes (S.I. Figure S12d). Dopa species show a rich follow‐up chemistry that could lead to crosslinking.^26^ However, the given conditions promote thiol‐Michael‐addition to dopaquinone moieties.^25^ The polymerization pathway was previously described and involved a complex redox‐interplay of thiol/disulfide and *o*‐quinone/*o*‐diphenols.^16^ MALDI‐TOF‐MS/MS analysis of poly([C/Y]‐(VT)_1_^Ψ9^) confirmed the polymerization mechanism by showing the conclusive fragments of cysteinyldopa such as thiol‐quinone adducts and α,β‐di‐dehydroalanine fragments (S.I. ). Moreover, no evidence was found suggesting the formation of alternative lysinyldopa‐linkages. SDS‐PAGE proved for all unimers [C/Y]‐(VT)^Ψ^ a rapid polymerization by showing no changes of polymer bands after 10–15 minutes (S.I. Figure S11). The Cys residue in the [C/Y]‐domain was essential for polymerization as activation of [S/Y]‐(VT)_5_^Ψ9,13,17^ failed to form polymers (S.I. Figure S11). GPC shows for all poly([C/Y]‐(VT)_*n*_^Ψ^) monomodal distributions with *Ð*_app._=1.4–1.5 and molecular weights of M_n,app._=28–32 kDa (S.I. Figure S13, Table S2). IR and CD spectroscopies show the integrity of the depsi‐segments within the polymers by confirming the presence of depsi‐ester bands at 1748 cm^−1^ and the absence of β‐sheets at pH 5.5 (Figure 2 a, S.I. Figures S9&S15).

**Figure 2:**
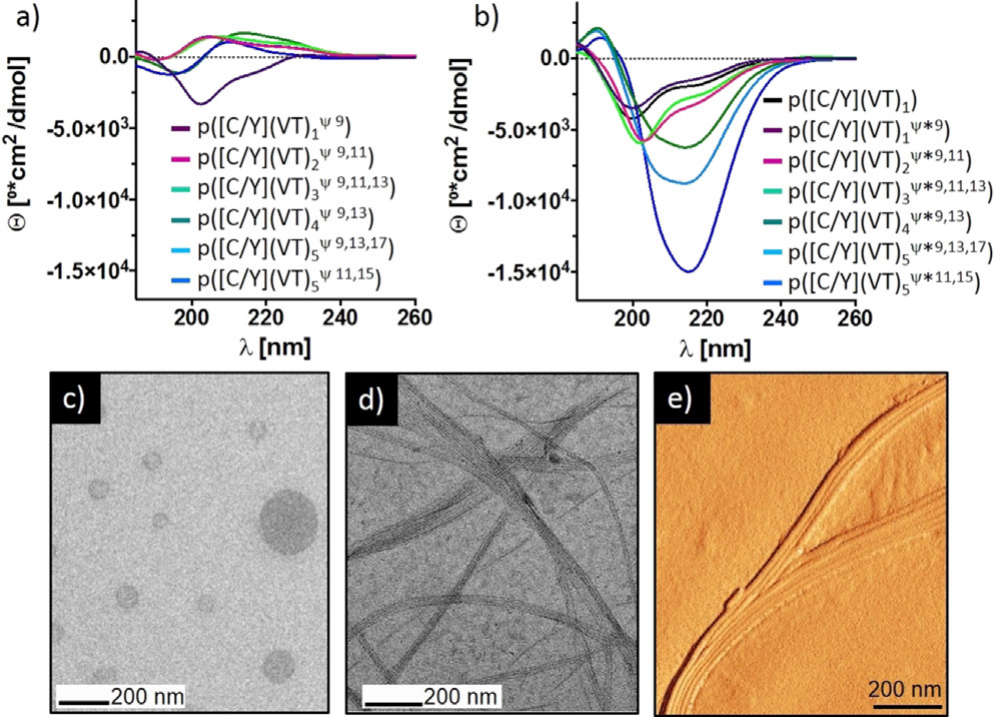
Activating β‐sheet formation in artificial mfps by pH changes. a, b) CD spectra of non‐switched (a; pH 5.5) and switched (b; pH 7.4) mfps suggesting a transition in secondary structure occurs. c, d) TEM images of poly([C/Y]‐(VT)_5_^Ψ11,15^ prior to (c) and after (d) switching indicate the formation of β‐sheet fibrils, which is confirmed by AFM micrographs of poly([C/Y]‐(VT)_5_^Ψ11,15^ after switching (e).

After adjusting the pH to 7.4, the O→N‐acyl transfer rearrangement took place in the switch segments of the polymers to restore the β‐sheet propensity of (VT)_*n*_ domains. This was evident by IR and CD analysis, showing no ester vibrations in the IR spectra and the typical CD Cotton effects for β‐sheets (+193 nm & −214 nm) (Figure 2 b, S.I. Figure S15). With increasing (VT)_*n*_ lengths of the polymerized unimers the β‐sheet Cotton effects gets more evident in the spectra and from (VT)_4_ segments onwards the β‐sheet signals dominate. However, intrinsically unstructured regions are still evident in the CD spectra. Those are expected, as [C/Y] domains with cysteinyldopa connectivities were unable to adapt undisturbed β‐sheets. Probably, the cysteinyldopa‐structures are providing high solubility and retard gel formation of switched poly([C/Y]‐(VT)_5_^Ψ*11,15^) compared to [C/Y]‐(VT)_3_, that gel at even lower concentration.

Consistent with IR and CD spectroscopy, TEM and AFM micro‐graphs show fibrillar aggregates to occur 8–24 h after switching poly([C/Y]‐(VT)_4_^Ψ9,13^) and poly([C/Y]‐(VT)_5_^Ψ11,15^) (Figure 2 d,e and S.I.). Those fibrillar structures were not formed in non‐switched states at pH 5.5, which confirms the effects of the depsi‐defects (Figure 2 c).

It can be expected that those structural transitions dramatically impact polymer properties for example adhesion and cohesion. Quartz crystal microbalance (QCM) measurements were carried out on Al_2_O_3_ coated sensors to reveal insight into adsorption kinetics and coating stabilities of artificial mfps prior to and after switching (S.I.). While the deposition of the non‐switched poly([C/Y]‐(VT)_5_^Ψ11,15^) at pH 5.5 reaches equilibrium rapidly, the non‐activated [C/Y]‐(VT)_5_^Ψ11,15^ unimers show negligible adsorption (S.I.). This was expected, as the unimers lack Dopa‐derivatives and the polymer acts as a polyelectrolyte. With +3 net charges per repeat unit (Lys12 + 2×depsi) Coulomb repulsion builds up, restricting mass deposition to coatings with 160 ng cm^−2^ as estimated by the Voight model.^27^ The properties changed significantly after switching as poly([C/Y]‐(VT)_5_^Ψ*11, 15^) can form at pH 7.4 β‐sheets and net charge per repeat unit is +1. The switched construct showed rapid adsorption with areal mass densities of 870 ng cm^−2^ (S.I. Figure S20). The coating withstands washes with hypersaline solution as found in the Dead Sea, leading to minor mass loss of 16 % (S.I. Figure S21)

The study on coating behavior was complemented by soft colloidal probe measurements^28^ analyzing underwater adhesion between silica surfaces at different pH and loading forces (S.I.). The poly([C/Y]‐(VT)_5_^Ψ11,15^) shows impressive changes of adhesion forces on silica at pH 5.5 and pH 6.8. The Johnson‐Kendall‐Roberts (JKR) model provides work of adhesion (*W*_adh_) from measured adhesion forces.^29^ At load force of 500 nn, work of adhesion increased by ≈490 % from *W*_adh‐pH5.5_=0.54±0.09 mJ m^−2^ to *W*_adh‐pH6.8_=2.64±0.15 mJ m^−2^ (Figure 3 a) and a similar trend was observable for 100 nn load force.

**Figure 3:**
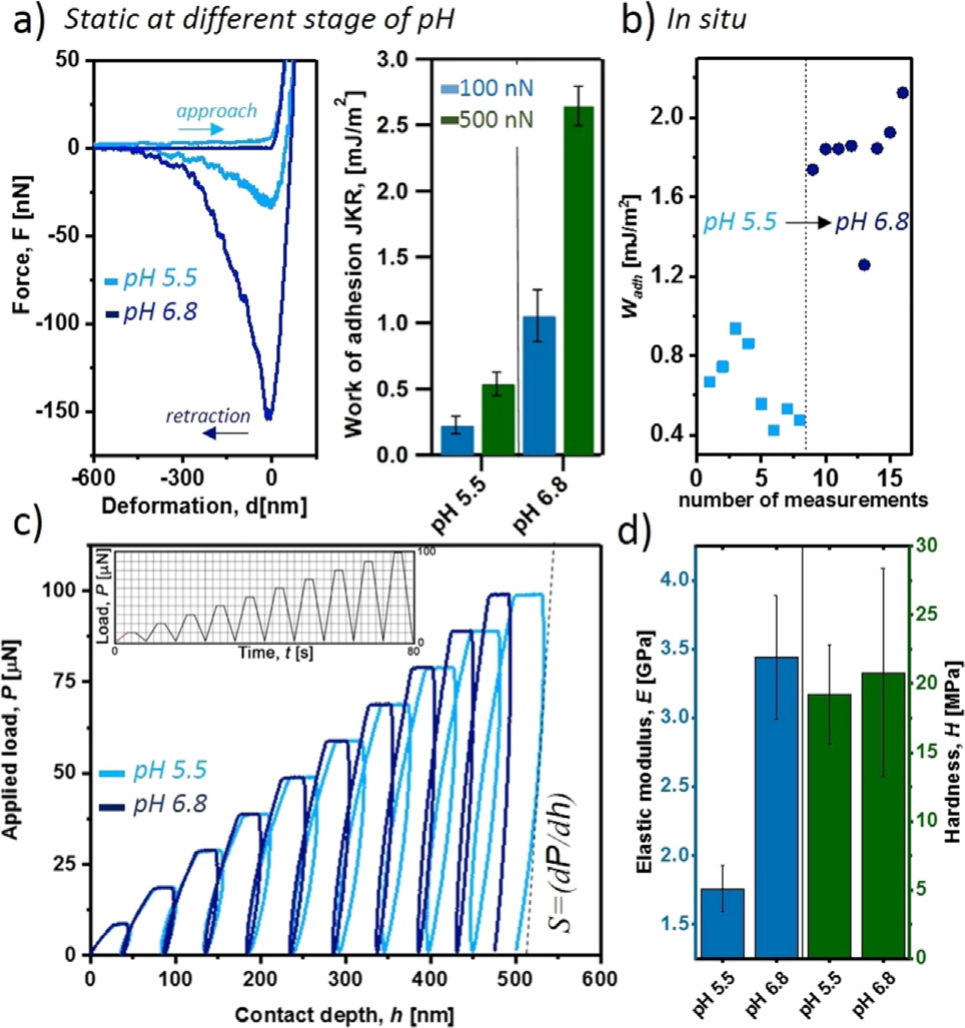
Adhesion and cohesion properties of poly([C/Y]‐(VT)_5_^Ψ11,15^ prior to and after switching. a) Adhesion measurements by soft colloidal probe AFM on glass with a PDMS probe in static experiments at different pH values. Force‐deformation curves at 500 nn loading (left) and pH‐dependent work of adhesion (right) are shown. b) In situ adhesion measurements with switching of the coating from pH 5.5 to 6.8. c, d) Mechanical properties obtained from depth‐sensing nanoindentation. Depth profiling cyclic load function (c) and extracted mechanical responses at different pH (d) are shown.

Adhesion energy differences in a related range were found also in in situ switch experiments, where adhesive forces have been mapped on the same coating prior and after the pH‐changes. The adhesive coating performs a notable transition from a moderate to a 300 % improved adhesive state (*W*_adh‐pH5.5_=0.60±0.19 mJ m^−2^ and *W*_adh‐pH6.8_=1.80±0.37 mJ m^−2^ for 500 nn load force, Figure 3 b and Figure S25). As known for strong adhesive coatings, the probe can collect some materials during the measurements, causing with increased contact numbers higher scattering (S.I.). This was not affecting the first half of the data set, providing rather constant *W*_adh_ values. The ultimate work of adhesion to silica was found in static experiments at pH 6.8. The remarkable value meets a similar range found for silica adhesion of a recently reported artificial mfp‐1^16^ and of isolated mfp‐3 & 5 that define the adhesive interface in mussels.^30^ Nonetheless, a quantitative interpretation of adhesive energy changes in the in situ experiments seems not to be trivial. Several effects are expected to superimpose with difficult to determine strengths of contributions. The change in pH from 5.5 to 6.8 modulates substrate surface potentials and triggers the depsi‐switch that changes both net charges in the polymer bulk and β‐sheet formation tendency. Those effects will stabilize the bulk network of the adhesive polymers by secondary structure formation and reduce Coulomb repulsion, which in turn might affect the surface contacts positively.

Obviously, the (VT)_*n*_ cohesion module does not dramatically interfere with the adhesive properties of poly([C/Y]‐(VT)_5_^Ψ*11,15^) at pH 6.8. A significant effect on mechanical bulk properties can be expected depending on the disturbed or activated β‐sheet structure formation. The elastic‐inelastic response was characterized on thick films cast from poly([C/Y]‐(VT)_5_^Ψ11,15^) at pH 5.5 or pH 6.8, using depth‐sensing nanoindentation (Figure 3 c). A significant increase in elastic modulus (*E*) form *E*_pH5.5_=1.76±0.17 GPa to *E*_pH6.8_=3.44±0.45 GPa revealed the dominant effect of the new β‐sheet linkages on the elastic response of the polymer bulk. The E‐moduli fall within the range of their biological counterparts as the distal byssus region of *M. californianus*, which contains preCol‐D with alanine‐rich β‐sheet domains reaches *E*=0.87 GPa and the protective distal cuticle shows *E*=0.5–2.2 GPa.^31^ Considering, *E*=1.2 GPa of mammalian collagen rich tendon and *E*≈5–12 GPa reached by *Bombyx mori* silk fibroin, the artificial mussel glue protein meets an important E‐moduli window of highly purpose adapted biomaterials.^32^ According to the Oliver‐Pharr method,^33^ the contact stiffness (*S*) correlates with the calculated E‐modulus (Figure 3 c). In contrast, the hardness (*H*), which denotes the inelastic response, was barely affected by β‐sheet structure formation (*H*_pH5.5_=19.23±3.61 MPa and *H*_pH6.8_=20.79±7.56 MPa). Such differentiated behavior in elastic‐inelastic response suggests that activated formation of β‐sheets promote the resistance of the bulk adhesive to cope with reversible/elastic deformations, confirming improved cohesion.

In conclusion, artificial mussel‐glue proteins were accessed by a tyrosinase activated polymerization of peptide unimers. Those exhibit [Tyr/Cys] modules for polymerization via cysteinyldopa linkage formation and (Val‐Thr)_*n*_ segments for cohesion control. The β‐sheet propensities of the latter were suppressed by depsi‐switch defects, enabling ease of polymerization and handling of the polymers. The β‐sheet formation could be activated by pH‐controlled rearrangement of the switch defects as shown by CD spectroscopy and microscopy. Programmed in the molecular structure the artificial mfps combine adhesion and cohesion properties. The adhesives form coatings on Al_2_O_3_ that defy hyper‐saline conditions, reach remarkable work of adhesion on silica of *W*_adh‐pH6.8_=2.64 mJ m^−2^ and show 200 % improved cohesive bulk stiffness through β‐sheet activation.

## Conflict of interest

The authors declare no conflict of interest.

## Supporting information

As a service to our authors and readers, this journal provides supporting information supplied by the authors. Such materials are peer reviewed and may be re‐organized for online delivery, but are not copy‐edited or typeset. Technical support issues arising from supporting information (other than missing files) should be addressed to the authors.

Supplementary
