# Supplementary material for: Toward Artificial Mussel‐Glue Proteins: Differentiating Sequence Modules for Adhesion and Switchable Cohesion
Source: Angew Chem Int Ed Engl. 2020 Aug 19;59(42):18495–9. doi: 10.1002/anie.202008515 (PMC7590116; doi:10.1002/anie.202008515)
Supplement: Supplementary file 1 — Supplementary [file ANIE-59-18495-s001.pdf]

Angewandte Chemie
Eine Zeitschrift der Gesellschaft Deutscher Chemiker

Supporting Information

# Toward Artificial Mussel-Glue Proteins: Differentiating Sequence Modules for Adhesion and Switchable Cohesion

*Sandra Arias, Shahrouz Amini, Justus Horsch, Matthias Pretzler, Annette Rompel, Inga Melnyk, Dmitrii Sychev, Andreas Fery, and Hans G. Börner**

anie_202008515_sm_miscellaneous_information.pdf

Supporting Information

©Wiley-VCH 2019
69451 Weinheim, Germany

## Table of Contents

**EXPERIMENTAL PROCEDURES** ........ **3**

1. MATERIALS ........ 3
   - *1.1 Peptide Synthesis* ........ *3*
   - *1.2 SDS-PAGE* ........ *3*
   - *1.3 Enzymatic assay and reactions* ........ *3*
2. INSTRUMENTATION ........ 4
3. METHODS ........ 6
   - *3.1 Peptide synthesis* ........ *6*
   - *3.2 Preparation of AbPPO4 tyrosinase from common mushrooms* ........ *6*
   - *3.3 AbPPO4 activity assay* ........ *6*
   - *3.4 Enzymatic unimer activation and polymerization reactions* ........ *7*
   - *3.5 SDS PAGE* ........ *7*
   - *3.6 Quartz crystal microbalance* ........ *7*
4. COMPOUNDS CHARACTERIZATION ........ 8
   - *4.1 MALDI characterization* ........ *8*
   - *4.2 Switch peptide integrity monitored by IR* ........ *13*
5. EXPERIMENTS ........ 13
   - *5.1 SDS PAGE polymerization kinetics* ........ *13*
   - *5.2 UV/vis measurements: pH control and enzyme activity* ........ *16*
   - *5.3 GPC measurements of peptides* ........ *17*
   - *5.4 Secondary interactions and switch behavior monitored by circular dichroism* ........ *17*
   - *5.5 Microscopy studies* ........ *19*
   - *5.6 QCM-D experiments on aluminum oxide surface* ........ *21*
   - *5.7 Soft colloidal probe atomic force microscopy to characterize adhesion properties* ........ *26*
   - *5.8 Depth-sensing nanoindentation measurements* ........ *29*

**REFERENCES** ........ **30**

## Experimental Procedures

### 1. Materials

Calcium chloride dihydrate (≥99.5%), sodium citrate tribasic dihydrate (>98%), potassium chloride (≥99.5%), α-cyano-4-hydroxy-cinnamic acid (α-CHCA, 99%) and formic acid (FA, ~98%) were purchased from Sigma Aldrich (Seelze, Germany). Acetonitrile (HPLC-MS grade), ethanol absolute (>99.7%) and hydrochloric acid (37%) were obtained from VWR chemicals (Philadelphia, USA). Citric acid (≥99.5%), sodium sulfate (≥99%), sodium hydrogen carbonate (≥99.5%), sodium bromide (≥99%) and strontium chloride hexahydrate (≥99%) were received from Carl Roth GmbH (Karlsruhe, Germany). Boric acid (99%) and magnesium chloride hexahydrate (98%) were purchased from abcr GmbH (Karlsruhe, Germany). Sodium chloride (≥99%) and potassium bromide (≥99%) were obtained from Acros Organics (Geel, Belgium). Hellmanex III was acquired from Hellma GmbH (Müllheim, Germany). All chemicals were used as received without further purification.

All buffers and aqueous solutions were prepared with Milli-Q water. Sodium citrate buffer was used for all experiments except for CD experiments where sodium phosphate buffer was used.

#### *1.1 Peptide Synthesis*

N-α-Fmoc protected amino acids Fmoc-Cys(Trt)-OH, Fmoc-Gly-OH, Fmoc-Lys(Boc)-OH, Fmoc-Ser(tBu)-OH, Fmoc-Thr(tBu)-OH, Fmoc-Tyr(Boc)-OH, Fmoc-Val-OH, Boc-Thr-OH, as well as coupling reagents 2-(1H-benzotriazol-1-yl)-1,1,3,3-tetramethyluronoium hexafluorophosphate (HBTU), Benzotriazole-1-yl-oxy-tris-pyrrolidino-phosphonium hexafluorophosphate (PyBOP), and N-methyl-2-pyrrolidone (NMP, 99.9%, peptide synthesis grade) were used as received from IRIS Biotech GmbH (Marktredwitz, Germany). Fmoc-Rink Amide resin 100-200 mesh (loading: 0.74 mmol/g) was obtained from Rapp Polymere GmbH (Tübingen, Germany). N,N-diisopropyl ethylamine (DIPEA, peptide grade), piperidine (peptide grade) and 2,5-dihydroxybenzoic acid (99%) were purchased from Acros Organics (Geel, Belgium) and used without further purification. Triethylsilane (TES, 98+ %) was obtained from Alfa Aesar (Karlsruhe, Germany), trifluoroacetic acid (TFA, peptide grade) came from Acros Organics (Geel, Belgium). Dichloromethane (DCM, peptide grade) from IRIS Biotech GmbH (Marktredwitz, Germany) was distilled from $CaH_2$ prior to use. The peptides were accessed in 80-100 mg each (0.1 mmol reaction scale)

#### *1.2 SDS-PAGE*

Dodecyl sulfate sodium salt (85%) was purchased from Acros Organics (Geel, Belgium). Glycine (≥99%) was obtained from Sigma Aldrich (Seelze, Germany). Tris(hydroxymethyl)aminomethane (Tris, ≥99.9%) was acquired from Carl Roth GmbH (Karlsruhe, Germany). The PageRuler prestained protein ladder (10 - 180 kDa), the Pierce lane marker non-reducing sample buffer and the Pierce silver stain kit were purchased from Thermo Fisher Scientific (Waltham, MA, USA). The protein ladder and the lane marker were stored at -20 °C.

#### *1.3 Enzymatic assay and reactions*

The enzyme *Ab*PPO4 was prepared as described earlier (cf. section 3.2).[1] Lyophilisates of the enzyme from sodium citrate buffer (50 mM, pH 6.8) were stored at -20 °C and dissolved in Milli-Q water prior to use. Enzyme solutions were stored at -20 °C as well. L-Tyrosine (≥98%) was obtained from Sigma Aldrich (Seelze, Germany) and L(+)-ascorbic acid (≥99%) was purchased from Carl Roth GmbH (Karlsruhe, Germany). 0.7 nmol/U ascorbic acid was used as antioxidant additive to prevent possible oxidations in the material solutions and stored at -20 °C either as solutions or as lyophilized powders.

## 2. Instrumentation

**Ultraviolet–visible spectroscopy (UV-Vis).** The enzymatic assay was carried out in a UV-Vis EonC Microplate Spectrophotometer with cuvette port (BioTek, Bad Friedrichshall, Germany) using quartz cuvettes.

**Matrix-Assisted Laser Desorption/Ionization (MALDI-TOF).** MALDI-TOF mass spectrometry was performed on an autoflex III smartbeam system (Bruker, USA) with matrix assisted laser desorption/ionization and time of flight detector. On the sample plate, 2 µL of sample were mixed with 1 µL matrix solution, consisting either of 7 mg/mL α-cyano-4-hydroxy-cinnamic acid (CHCA) or 10 mg/mL 2,5-dihydroxybenzoic acid (DHB) in MQ-water-acetonitrile (1:1, v/v) with 0.1 % TFA. Samples were air-dried at ambient temperature. Measurements were performed in linear positive mode. Gating and deflection modes were used for detection of the higher mass area (m/z > 10.000).

**MALDI-TOF MS/MS** measurements were carried out on a MALDI-TOF/TOF 5800 system (AB Sciex, USA). As matrix α-cyano-4-hydroxycinnamic acid (HCCA, 10 mg $mL^{-1}$) was used.

**High-performance liquid chromatography (HPLC**). Analytical HPLC was performed on a Shimadzu (Japan) system using a SCL-10A vp system controller, a SPD-M10A vp diode array detector, a LC-10AD vp liquid chromatograph pump unit and a CTO-10AC vp column oven equipped with a YMC-Pack ODS-AQ column (250 x 4.6 mm, YMC, Japan). For preparative HPLC a Shimadzu prominence system with a CBM-20A communications bus module, a LC-20AP preparative liquid chromatograph pump unit, a SPD-10A UV/VIS detector and a FRC-10A fraction collector was used. Chromatographic separation was conducted on an XBridge BEH C18 OBD column (150 x 19 mm, Waters, Milford, USA). As solvent, mixtures of solvent A/solvent B (solvent A: 99.9% Milli-Q $H_2O$ - 0.1% FA; solvent B: 99.9% acetonitrile - 0.1% FA) were used for both systems with a flow rate of 1.0 and 22.0 mL/min respectively.

**Ultra-performance liquid chromatography coupled with a tandem quadrupole mass spectrometer (UPLC-QMS).** UPLC-QMS was carried out on an Acquity UPLC H-class system (Waters, USA) with a PDA and QDa detector. Acquity UPLC BEH C18 columns (2.1 x 100 mm, 2.1 x 50 mm, Waters, USA) were used for separation with a solvent mixture of solvent A/solvent B (solvent A: 99.9% Milli-Q $H_2O$ - 0.1% FA; solvent B: 99.9% acetonitrile - 0.1% FA) and flow rates of 0.5 mL/min.

**SDS-PAGE method**. SDS-PAGE measurements were performed in a Mini-PROTEAN tetra system cell (Bio-Rad, USA) with purchased 4 - 20% gel percentage precast polyacrylamide Mini-PROTEAN TGX gels (Bio-Rad, USA). As running buffer a solution of 25 mM Tris, 192 mM glycine, 0.1% SDS in Milli-Q water was used. Staining was done according to standard protocol with a Pierce silver stain kit (Thermo Fisher Scientific, USA).

**Circular dichroism spectroscopy.** CD measurements were done in a Jasco-720 with a Hellma™ quartz cuvette with 1.0 mm path length and 350 µL fill volume. The amounts of polymer used for CD measurements were 250 µM in phosphate buffer (10 mM) at different pH and T=25°C. The results are shown in molar ellipticities Θ.

**Fourier transformation infrared spectroscopy (FT-IR)**. FT-IR spectra were recorded on a Bruker Vertex 70v FT-IR spectrometer (Bruker Optics GmbH, Ettlingen, Germany) with an evacuable optics bench in a range from 4000-400 $cm^{-1}$. Samples were measured in solid form in ATR-IR modus under vacuum system.

**Gel permeation chromatography (GPC)**. Aqueous GPC was carried out on a NOVEMA Max analytical linear XL column (PSS, Germany) calibrated with pullulanes with an AS-100 autosampler, P-100 pump (TSP Thermo Separation Products, Germany) and Shodex RI-101 detector (VDS-optilab, Germany). As mobile phase acetate buffer (100 mM, pH 4.5): methanol, 4:1 (v/v) was used with a flow rate of 1.0 mL/min. Data were recorded and evaluated with the PSS-WinGPC Unichrom software package.

**Quartz crystal microbalance (QCM)**. QCM measurements were conducted on a Q-sense Explorer E1 single-sensor QCM-D module with dissipation combined with a QE 401 Electronic Unit (Biolin Scientific, Sweden) and equipped with an IPC-N 4

multichannel pump (Ismatec, Germany). Piezoelectric sensor crystals coated with 100 nm aluminum oxide (QSX 309, Biolin Scientific, Sweden) were used for adsorption measurements.

**Transmission electron microscopy (TEM)**. 10µL of 0.5 µM of samples were spotted on a carbon film 300 mesh, copper grid. TEM imaging was performance using Talos TEM from Field electron and ion (FEI).

**Atomic force microscopy (AFM).** For atomic force microscopy, the samples were spin-coated (3000 rpm) from solution (0.5-0.05 µM) on freshly cleaved Mica substrates. AFM imaging was done in tapping mode under a silicon nitride cantilever using Veeco Nanoscope VIII Multimode AFM. Commercial silicon tips (Type SCANASYST-AIR) were used with a tip radius 2-12 nm, employing a spring constants 0.4 $Nm^{-1}$ at a resonance frequency of 50-90 kHz.

For adhesion measurements, clean, plasma activated (0.2 mbar, 100W, 1 min; 440-G, TePla, Wettenberg, Germany) glass slides were coated with the polymers and an AFM (MFP 3D, Asylum Research, Oxford Instruments, California, USA) were used a soft colloidal probe was chosen for the adhesion measurements, because this results in a significantly larger contact area than for hard colloidal probes (e.g. glass, silica). Adhesion experiments were performed with a tipless cantilever (NSC 35, Mikromasch Europe, Wetzlar, Germany) equipped with a Polydimethylsiloxane (PDMS) soft colloidal probe (diameter 23.6 µm, E-modulus ~ 2.2 MPa). Prior to use the cantilever was $O_2$ plasma treated (0.2 mbar, 100 W, 10 sec; 440-G, TePla, Wettenberg, Germany) to activate the bead surface and establish reproducible conditions. Determination of the spring constant of the cantilever was achieved by measurement of thermal noise[2](k=15.4 N/m) and by pressing the cantilever against a non-deformable surface lever sensitivity[3] was determined for the tipless cantilever. Since lever sensitivity with a soft colloidal probe cannot be calculated by the same method, thermal noise was used to assess this value. The sensitivity of the soft colloidal probe cantilever is in good agreement with the tipless one.

For measurements on in-situ switching of adhesion the PDMS colloidal probe had diameter of 26.6±0.2 µm and E-modulus ~ 2.0 MPa. The soft colloidal probe was attached to a tipless cantilever with k=16.6 N/m. The diameter of soft colloidal probe was measured with an optical microscope.

**Nanoindentation**. Nanoindentation studies were done using a TriboIndenter TI-950 (Hysitron-Bruker, MN, USA) equipped with a standard 2D transducer and a Berkovich tip. The tip was calibrated for the required contact depths using a standard PMMA sample (E=5.13 GPa). A cyclic load function composed of 10 cycles with an increment of 10 µN/cycle and a max. load of 100 µN was used for measurements. The cyclic load function was used to make sure that the extracted indentation curves were not affected by the silicon wafer substrate or any inhomogeneity on the surface of the films. The Oliver-Pharr method[4] was used for calculation of the elastic modulus and hardness of the samples. Scanning probe microscopy was used to measure the thickness of the samples (tpH 5.5 = 8 µm and tpH 6.8 = 100 µm), which were more than 10 times thicker than the maximum contact depths.

## 3. Methods

### 3.1 Peptide synthesis

Peptides were synthesized following standard ABI-Fastmoc protocol (single coupling with capping) with NMP as solvent using standard Fmoc-amino acid derivatives. As solid support, Fmoc Rink Amide resin (loading 0.74 mmol/g, 0.1 mmol, 100-200 mesh) was used. Synthesis was performed on an automated ABI 433a peptide synthesizer (Applied Biosystems, Foster City, USA). Fmoc-amino acid coupling was facilitated by HBTU/DIPEA. After final Fmoc removal, the resin was transferred to a 10 mL syringe reactor and subsequently washed with dichloromethane. Peptides were cleaved from the solid support with a mixture of 95:4:1 vol.% TFA/$H_2O$/TES for 3 h, which resulted in fully deprotected peptide. In the case of peptides whose C-terminal is a Boc-AA, the cleavage from the solid support (3 ml/10μmol) was carried out with a mixture of 80:15:5 vol.% TFA/TFMSA/m-cresol for 2 h. In both cases, the resin was filtered, washed with TFA and the collected supernatants were concentrated in vacuo. The product was isolated by precipitation with diethyl ether and subsequent centrifugation. Purified products were obtained by lyophilization from Milli-Q water or from Milli-Q water with 0.1% HCOOH for peptides that contains switch segments.

3.1.1 Synthesis of the switch segment -Val(Boc)Thr-

The coupling of the Fmoc-Val-OH onto the unprotected hydroxyl side chain functionality of the prior attached Boc-Thr-OH was accomplished in a syringe reactor in NMP. Coupling was facilitated by Fmoc-Val-OH/DIC/NMI 10/10/7.5 eq. and repetitive coupling cycles were carried out to force the reaction to completion (at least 3×2 h and 1×12 h). The resin was washed with NMP several times after every coupling step. The last coupling was followed by a capping step (10% DIPEA, 10% Ac2O in NMP, 2×10 min) and Fmoc deprotection. Afterwards the resin was washed with NMP, and peptide synthesis was continued as described above.

### 3.2 Preparation of AbPPO4 tyrosinase from common mushrooms

The gene encoding *Ab*PPO4 was PCR-amplified from cDNA derived from an A. bisporus fruiting body at growth stage 5[5] and cloned into the expression vector pGEX-6P-1 (GE Healthcare Europe, Freiburg, Germany). The resulting construct encoding glutathione S-transferase [6] N-terminally fused to *Ab*PPO4 was expressed in E. coli BL21(DE3) grown in LB media supplemented with 2 mM $MgSO_4$, 500 mM NaCl, 1x mineral stock M[7], 1x sugar stock 5052, 100 mg $l^{-1}$ Na-ampicillin and 0.5 mM $CuSO_4$ at 20 °C for approximately 40 h. Cells were lysed by high-pressure extrusion and non-target proteins were removed by affinity chromatography on Glutathione Sepharose (GE Healthcare). The fusion partner GST was removed by proteolysis with GST-tagged picornain 3C which was afterwards removed along with the cleaved-off GST by a second round of affinity chromatography on the same column material. The resulting latent *Ab*PPO4 was activated by limited proteolysis with proteinase K and the active *Ab*PPO4 was purified by size exclusion chromatography on a Superdex 200 Increase column (GE Healthcare).

### 3.3 AbPPO4 activity assay

Prior to use of enzyme, an activity assay was performed using UV spectroscopy based on the method of Duckworth and Coleman.[8] The absorbance from the oxidation of tyrosine to Dopaquinone is monitored at 280 nm over a period of 20 min at 25 °C using a 3 mL quartz cuvette. The assay solution contained 1 mL of sodium citrate buffer (50 mM, pH 6.8), 1 mL of tyrosine solution (1 mM in Milli-Q water), 0.9 mL of Milli-Q water and 0.1 mL of *Ab*PPO4 enzyme solution (in 50 mM sodium citrate buffer, pH 6.8). Enzyme solution was added immediately before starting the measurement. The activity was calculated using the average slope of the 3 minute interval ΔA280 with the maximum slope of the absorbance-time curve according to equation S1.

$$\text{volumetric enzyme activity}\left[\frac{Units}{mL}\right]=\frac{\Delta_{\mathrm{A280[min^{-1}]}}}{0.0001} \qquad \text{(eq. S1)}$$

### 3.4 Enzymatic unimer activation and polymerization reactions

Standard enzymatic activation reactions were performed using a substrate concentration of 0.25 µmol/mL (from 1.0 mM stock solutions) and 100 U/mL of *Ab*PPO4 tyrosinase in a sodium citrate buffer solution (17 mM, pH 5.5) at 25 °C. AbPPO4 was mixed with 0.7 nmol/U ascorbic acid as an additive for the enzyme prior to addition to the substrate solution. In polymerization reactions for GPC, TEM, AFM, nanoindentation and QCM experiments, 0.75 µmol/mL substrate concentration and 50 U/mL *Ab*PPO4 were used.

### 3.5 SDS PAGE

Samples for gel electrophoresis as well as the protein ladder were diluted with Milli-Q water to a volume of 20 µL and subsequently 5 µL of lane marker were added and mixed. The gel cassettes were clamped into the electrode assembly and the assembly and the tank were filled with approximately 800 mL of running buffer (25 mM Tris, 192 mM glycine, 0.1% SDS in Milli-Q water). Samples of 20 µL were loaded into the wells of the gel using a 10-100 µL pipette and runs were performed at 140 V until the lane marker reached the lower end of the gel. Staining was performed using silver stain (Thermo Fisher Scientific, USA) according to the manufacturer's standard protocol.

### 3.6 Quartz crystal microbalance

The piezoelectric sensor crystals coated with 100 nm aluminum oxide (QSX 309, Biolin Scientific, Sweden) were cleaned with 2% Hellmanex III solution (in Milli-Q water) for 15 - 30 min and ethanol (absolute, >99.7%) in an ultrasonic bath for 10 min prior to use. Subsequently, the sensors were thoroughly washed with Milli-Q water and dried under a compressed air flow. Finally, the aluminium oxide coated crystals were cleaned by air plasma in a nanoETCH (Moorfield Nanotechnology) for 30 min 30W. The sensors were mounted into the QCM flow chamber and incubated with degassed sodium citrate buffer using a flow rate of 100 µL/min until the frequency signals were constant (1-3 h). The measurement was started and sample solutions were pumped into the flow chamber. Experiments were performed at 22 °C in a stop-flow mode, and overtones 3, 5, 7, 9, 11 and 13 were recorded. The third overtones of all experiments were used for evaluation of the frequency shift.

## 4. Compounds characterization

### *4.1 MALDI characterization*

- *Unimer: [C/Y]-(VT)$_1$*

A solution of unimer [C/Y]-(VT)$_1$ was activated according to the protocol given in 3.4 at pH 6.8. Figure S1b shows polymerization of [C/Y]-(VT)$_1$ after 15 min with a DP of up to 21 measured using CHCA matrix..

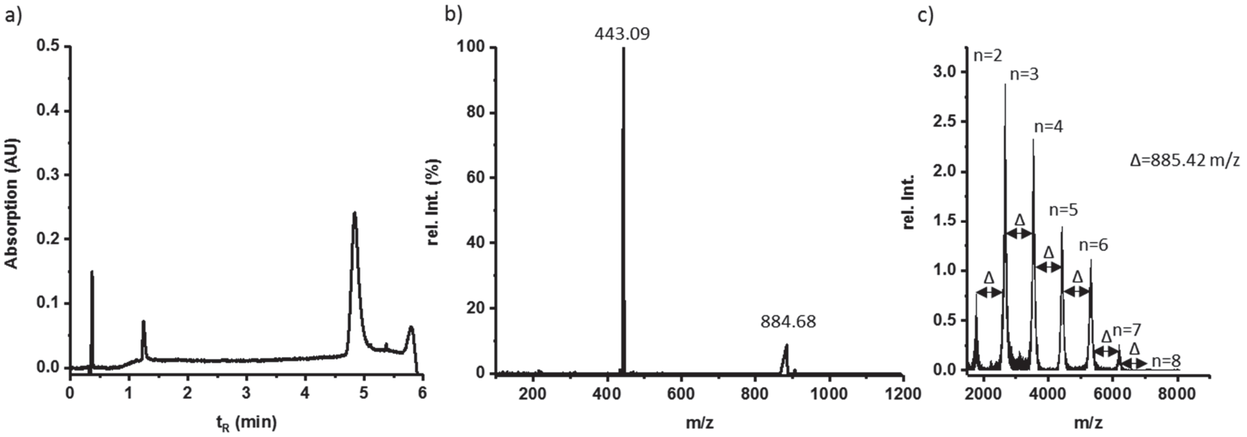


**Figure S1**. a) UPLC-UV/VIS-QMS: Solvent A: MQ-water, 0.1% FA, Solvent B: acetonitrile, 0.1% FA, gradient 5-25% B (6 min). UV/VIS: $t_R$ = 1.25 min, 96.33% purity. b) ESI-QMS: calculated: $[M+H]^+$ = 885.41, $[M+2H]^{2+}$ = 443.20; found: $[M+H]^+$ = 884.68, $[M+2H]^{2+}$ = 443.09. c) MALDI-TOF-MS spectrum of poly([C/Y]-(VT)$_1$) 15 min after enzymatic oxidation.

- *Switch Block Unimers by MALDI-TOF-MS*
- *[C/Y]-(VT)$_1^{\psi\ 9}$*

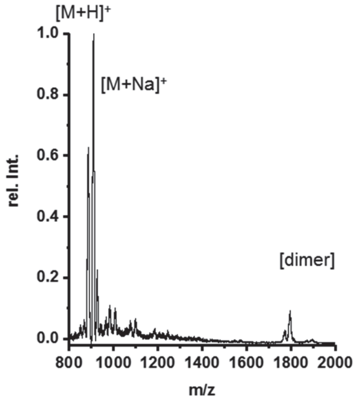


**Figure S2.** MALDI-TOF-MS analysis of [C/Y]-(VT)$_1^{\psi\ 9}$. Calculated: M = 885.63 Da, found: $[M+H]^+$ = 885.6 Da, $[M+Na]^+$ = 908.52 Da, $[M+M]^+$ = 1771.22 Da.

- *[C/Y]-(VT)$_2^{\psi\ 9,11}$*

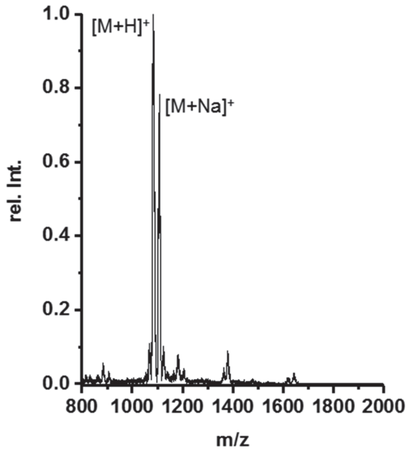


**Figure S3**. MALDI-TOF-MS analysis of [C/Y]-(VT)$_{2}^{\psi\,9,11}$ Calculated: M = 1084.84 Da, found: $[M+H]^+$ = 1085.6 Da, $[M+Na]^+$ = 1108.55 Da.

- *[C/Y]-(VT)$_{3}$ $^{\psi\,9,11,13}$*

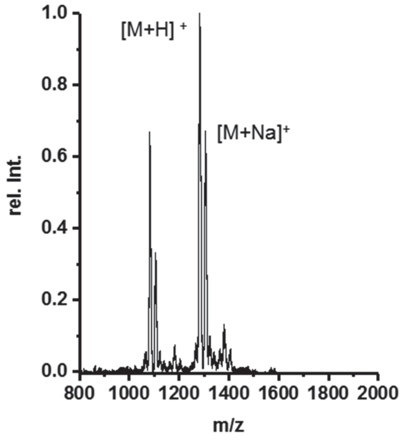


**Figure S4**. MALDI-TOF-MS analysis of [C/Y]-(VT)$_{3}^{\psi\,9,11,13}$. Calculated: M = 1284.63 Da, found: $[M+2H]^+$ = 1286.2 Da, $[M+Na]^+$ = 1308.09 Da.

- *[C/Y]-(VT)$_4$ $^{\psi\ 9,13}$*

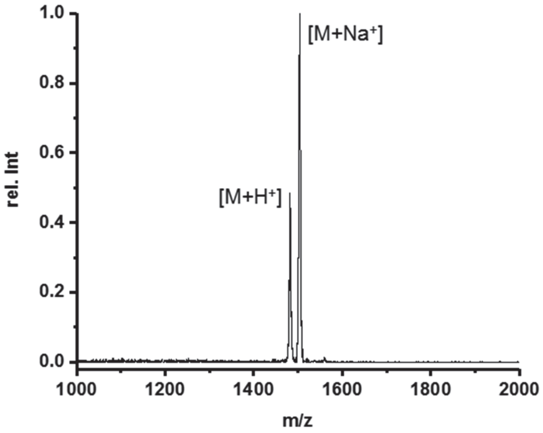


**Figure S5.** MALDI-TOF-MS analysis of [C/Y]-(VT)$_4$ $^{\psi\ 9,13}$. Calculated: M = 1484.63 Da; found: $[M+H]^+$ = 1486.96 Da, $[M+Na]^+$ = 1508.37 Da.

- *[C/Y]-(VT)$_5$ $^{\psi\ 11,15}$*

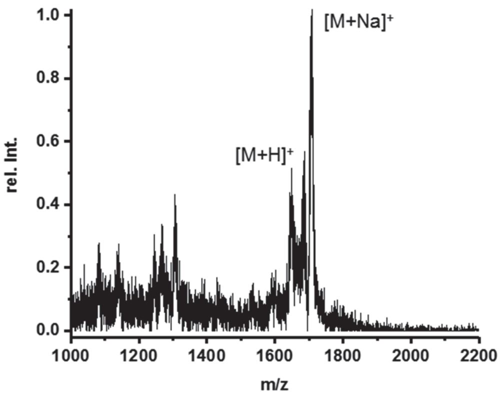


**Figure S6.** MALDI-TOF-MS analysis of [C/Y]-(VT)$_5$ $^{\psi\ 11,\ 15}$. Calculated: M = 1658.4 Da; found: $[M+H]^+$ = 1658.97 Da, $[M+Na]^+$ = 1708.99 Da.

- *MALDI-TOF-MS/MS measurements of poly ([C/Y]-(VT)$_1$$^{\psi 9}$)*

Tandem MS fragmentation spectra were recorded for tyrosinase activated polymerization products of [C/Y]-(VT)$_1$$^{\psi 9}$. A solution of [C/Y]-(VT)$_1$$^{\psi 9}$ (0.25 mM) was polymerized for 10 min according to the protocol given in section S3.4 using 100 U/mL *Ab*PPO4 tyrosinase. 0.9 µL of 6 M HCl were added to 100 µL of polymerization solution to stop the enzymatic reaction, the solution was lyophilized and redissolved in ACN/water (1:1, v/v) with 0.1% TFA for MALDI-TOF-MS/MS analysis. MALDI-TOF-MS/MS shows the typical homologues row, having the unimer mass differences as expected (Figure S7b). The signals corresponding to the dimer were found at m/z 1790 - 1796 (Figure S7b, and S7c inset). In the resulting fragmentation spectrum 75% of the corresponding y- and b-ions for the dimer could be found (Table S1). None of the signals indicated the presence of lysinyldopa adducts, which could potentially occur in a much slower alternative reaction pathway.[9] Instead, fragment **1** and fragment **2** were observed, resulting from S-C$_\beta$ bond cleavage of the cysteinyldopa linked [C/Y]-(VT)$_1$$^{\psi 9}$ dimer (Figure S8).

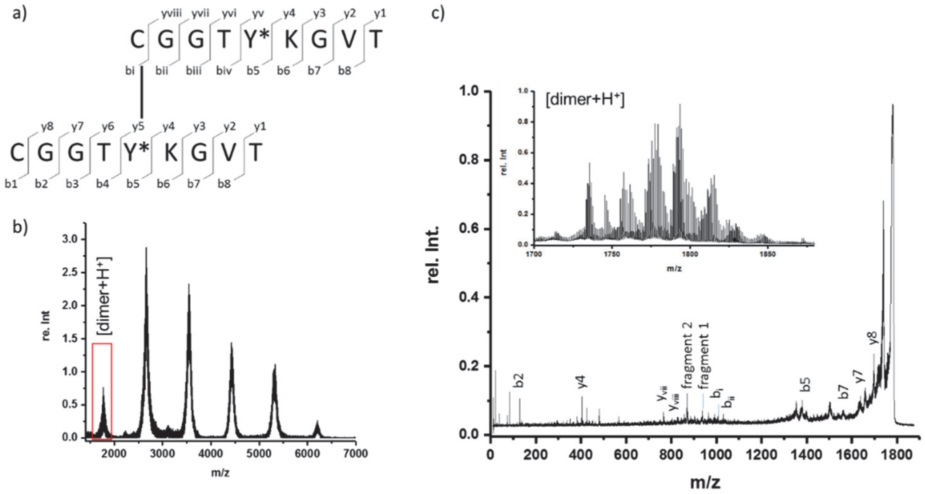


**Figure S7**. a) Assignment of the fragmentation ions of the cysteinyldopa linked [C/Y]-(VT)$_1$$^{\psi 9}$ dimer. b) MALDI-TOF-MS spectrum of poly[C/Y]-(VT)$_1$$^{\psi 9}$. c) MALDI-TOF-MS/MS fragmentation spectrum of the poly[C/Y]-(VT)$_1$$^{\psi 9}$ dimer (Inset shows the parent MS spectrum).

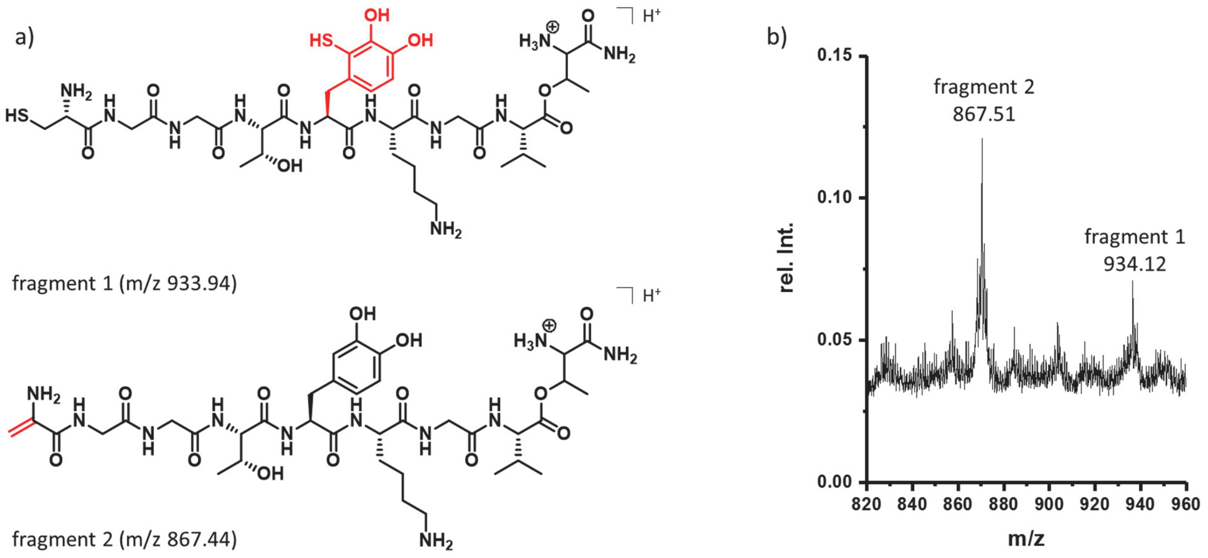


**Figure S8**. a) Structures of fragmentation ions 1 and 2, directly resulted from a bond break of poly([C/Y]-$(VT)_1^{\psi 9}$). b) Close-up of the MALDI-TOF-MS/MS spectrum showing fragmentation ions 1 (m/z 934.12) and 2 (m/z 867.51).

**Table S1.** List of ions found in MALDI-TOF-MS/MS analysis of the [dimer +H$^+$] mass from poly ([C/Y]-$(VT)_1^{\psi 9}$). Shaded ions are characteristic for a cysteinyldopa cross-link.

| ion | calc. [Da] | found [Da] | ion | calc. [Da] | found [Da] |
|---|---|---|---|---|---|
| b1 | 104.02 | - | y1 | 120.06 | 121.01 |
| b2 | 163.75 | 164.09 | y2 | 218.13 | 218.12 |
| b3 | 219.07 | 220.12 | y3 | 276.15 | 276.84 |
| b4 | 321.92 | 322.21 | y4 | 403.24 | 403.32 |
| b5 | 1398.56 | 1398.87 | y5 | 1481.69 | - |
| b6 | 1522.65 | - | y6 | 1581.71 | - |
| b7 | 1583.68 | 1584.11 | y7 | 1637.76 | 1638.03 |
| b8 | 1678.75 | - | y8 | 1696.98 | 1697.15 |
| $b_i$ | 1004.81 | 1005.05 | $y_v$ | 582.30 | 582.32 |
| $b_{ii}$ | 1058.43 | 1058.98 | $y_{vi}$ | 685.15 | 685.30 |
| $b_{iii}$ | 1115.45 | - | $y_{vii}$ | 740.87 | 741.17 |
| $b_{iv}$ | 1219.50 | 1220.11 | $y_{viii}$ | 798.39 | 798.58 |

.

## 4.2 Switch peptide integrity monitored by IR

The evidence of the rearrangement of the switched peptides to their native backbone were collected by infrared analyses. The presence of the switch ester carbonyl bands (Osec-ester band at v ≈ 1748 $cm^{-1}$) of the depsipeptides are visible and stable (Figure S9b). After restoring the native backbone by minor pH changes from pH 5.5 to pH 6.8, the ester carbonyl of the depsipeptide disappears and the carbonyl vibration band of the ester group is no longer observed in the infrared spectra (Figure S9c). In polymers case the presence/absence of the carbonyl band of the ester group by pH control is also observed (Figure S9d).

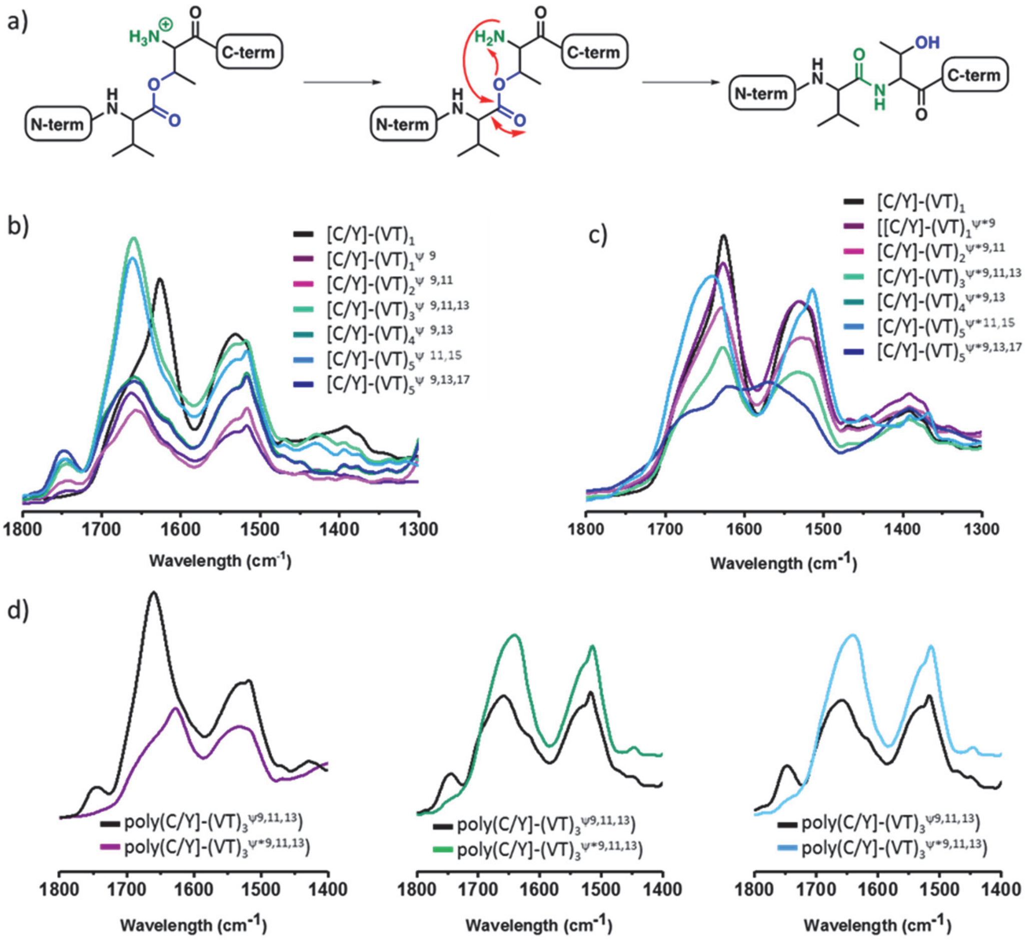


**Figure S9**. a) Illustration of the O→N-acyl rearrangement in switch segments by pH changes. ATR-IR measurements of depsipeptides a) before and b) after switch. d) ATR-IR measurements of polymers with longer $(VT)_n$ segment in the sequence.

## 5. Experiments

### 5.1 SDS PAGE polymerization kinetics

A 0.25 mM solution of [C/Y]-$(VT)_{1-3}$ was polymerized according to the protocol given in the section 3.4 using 50 U/mL *Ab*PPO4 tyrosinase. Due to the absence of the depsi-switches the pH of the buffer solution used was 6.8. Samples of 20 µL were taken after different reaction times and 0.5 µL of 3.6 M HCl were added (resulting in pH 2 of the sample solution) to stop the enzymatic reaction. Samples were taken from the reaction mixture after ~30 minutes. Subsequently the samples were frozen in liquid

nitrogen and stored at -20 °C until SDS PAGE measurement. 15 µL of each reaction sample and an *Ab*PPO4 tyrosinase reference were loaded onto the gel for measurements (Figure S10 and S11).

Depending on the length of the cohesion module, strong interferences with the enzymatic activation and polymerization process were evidenced. Due to the high tendency of (VT) domains to form β-sheets, the unimers and polymerization products was difficult. While [C/Y]-$(VT)_1$ could be rapidly activated by *Ab*PPO4 and polymerization leads to an intense band at ~35 kDa, [C/Y]-$(VT)_2$ directly forms gels during polymerization, and the peptide [C/Y]-$(VT)_3$ shows gel formation prior to polymerization unable to perform the enzymatic oxidation (Figure S10).

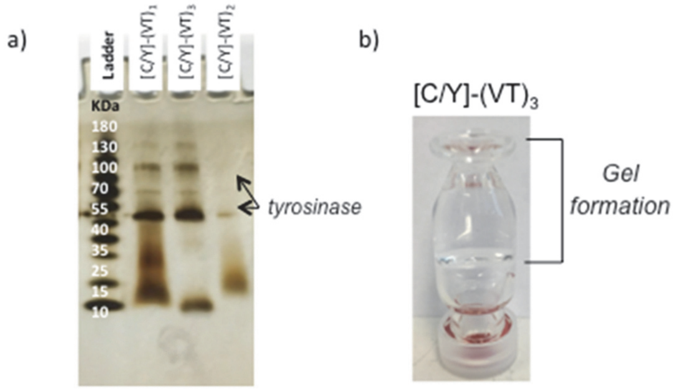


**Figure S10**. SDS PAGE measurement of polymer reaction after 30 min. of the different unimers, 50 U/mL *Ab*PPO4 tyrosinase. b) Photograph of a vial showing the gel formation of the peptide ([C/Y]-/$VT)_3$.

On the other hand, the unimers containing depsipeptide connectivities suppress the aggregation tendency of the (VT)n domains and the polymerization process was used according to the protocol given in the section 3.4. Figure S11 shows immediate formation of multimers ranging from ~10 kDa to ~35 kDa in apparent molecular weight. After 15 min reaction time the average molecular weight shifts to slightly higher values and an intense band at ~35 kDa is observed for poly([C/Y]-(VT) $_5^{\Psi 11,15}$). Further, no significant changes in molecular weight distribution are observed. This indicates a rapid polymerization process that leads to high yields after several minutes at an *Ab*PPO4 concentration of 50 U/mL. Additionally, stained area is visible at the bottom of the wells, which is not the case for the enzyme reference or the protein ladder. This indicates formation of high molecular weight material that cannot be resolved in the polymer matrix due to its molecular weight cutoff.

Peptide unimers having cysteine exchanged by serine residues were synthesized ([S/Y]-(VT)$_5^{\Psi9,13,17}$). The activation of [S/Y]-(VT)$_5^{\Psi9,13,17}$ by tyrosinase was performed under the same conditions as for [C/Y]-(VT)$_5^{\Psi9,13,17}$ but does in contrast not provide polymer products. This control experiment confirmed that enzymatic polymerization requires Cys residues to take place, which supports the pathway through cysteinyldopa linkages (Figure S11).

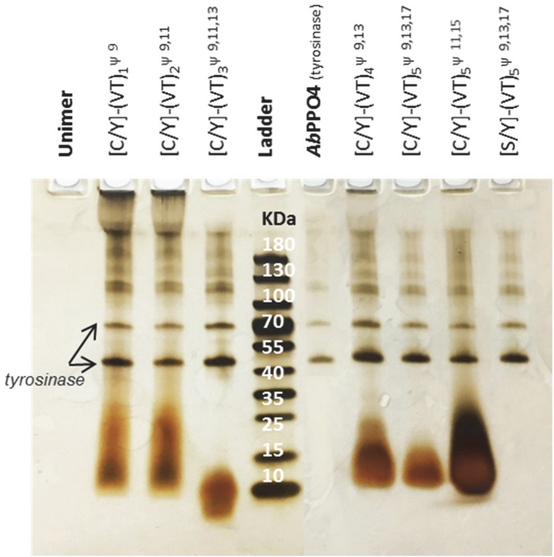


**Figure S11**. SDS PAGE measurement of unimers and tyrosinase as reference and the different polymer depsipeptides, after 15 min. 50 U/mL *Ab*PPO4 tyrosinase.

## *5.2 UV/vis measurements: pH control and enzyme activity*

It is known that tyrosinase enzyme has the highest activity at pH 6-7 in aqueous buffer solution. However, to prevent the high tendency of (VT) domains to form β-sheets, the enzymatic oxidation process has to carry out under mildly acid conditions. Therefore, a different enzyme oxidation process was carried out at different acid pH to determine the highest activity of the tyrosinase in these new conditions (Figure S12b). In buffered solution at pH 5.5 the enzyme proved to practically instantaneously oxidize the tyrosine residues of all [C/Y]-$(VT)_n^{\Psi}$ unimers to Dopa and Dopa quinone as shown by UV/vis spectra (Figure S12c). Only minor differences in the initial activation rates can be found as the rates increase consistently from [C/Y]-$(VT)_1^{\Psi 9}$ to [C/Y]-$(VT)_5^{\Psi 11,15}$. Interestingly, no significant differences in activation kinetics were evident if [C/Y]-$(VT)_1^{\Psi 9}$ was compared to [C/Y]-$(VT)_1$. However, the activation kinetics decrease as the switch defects of depsi-segments increase in number indicating that the positive charge of the depsi-segments interfere with the tyrosinase substrate properties of the unimers (Figure S12c).

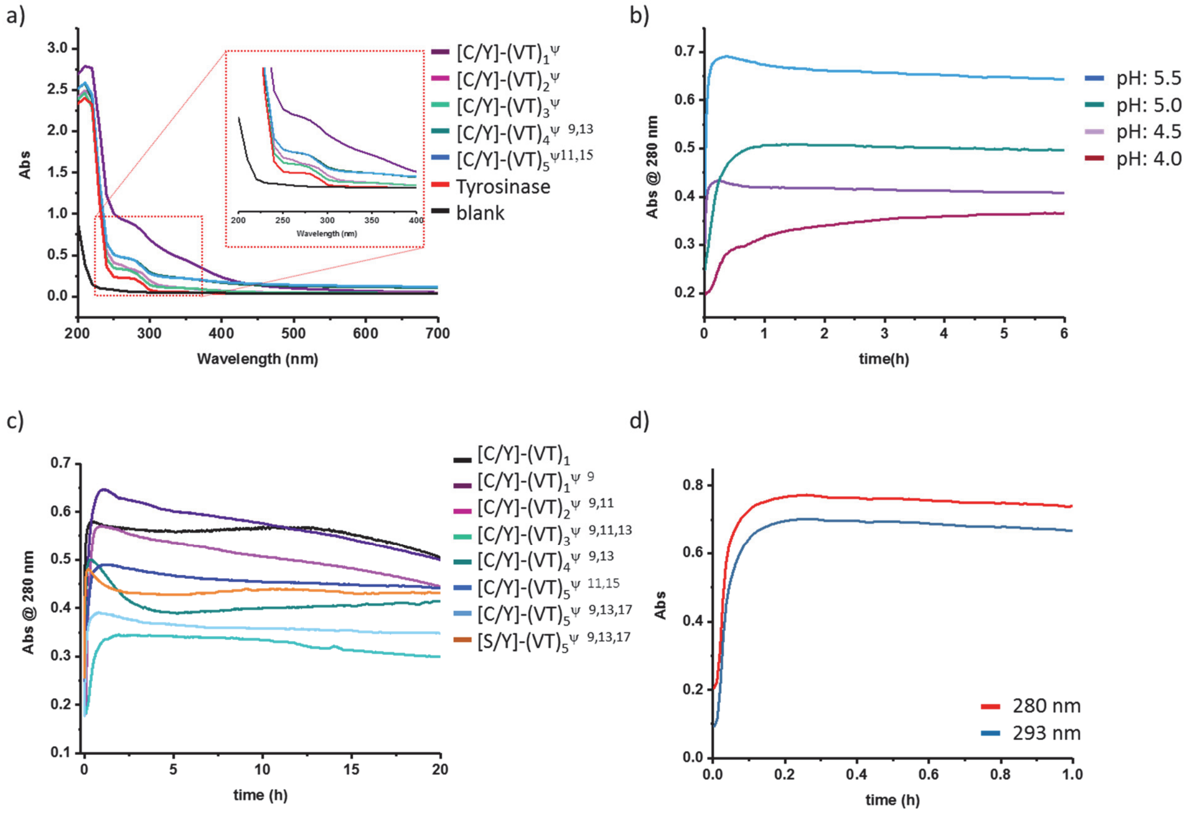


**Figure S12**. UV measurements of a) different depsipeptides in all spectra window at pH 5.5; b) enzyme activity in different mildly acid conditions at 280 nm; c) enzyme activity of each depsipeptide and the peptide[C/Y]-$(VT)_1$ at pH 5.5, 280nm; d) UV/vis monitoring of the enzymatic activation of [C/Y]-(VT)1 by tyrosinase (280 nm, and 293 nm, which are representative of Dopa, and cysteinyldopa).

## *5.3 GPC measurements of peptides*

A 0.75 µM solution of each depsipeptide was polymerized according to the protocol given in the section 3.4 using an *Ab*PPO4 tyrosinase concentration of 50 U/mL in 1.5 mL. After 30 minutes, 5 µL of 6 M HCl were added (resulting in pH 2 of the sample solution). Afterwards, samples were frozen in liquid nitrogen and lyophilized. For GPC measurement the lyophilized samples were completely redissolved in 2 mL Milli-Q water yielding 0.75 mg/mL polymer. All of the GPC measurement results are in accordance with SDS PAGE measurements (see Figure S13) where the polymers [C/Y]-(VT)$_1^{\Psi\ 9}$, [C/Y]-(VT)$_2^{\Psi\ 9,11}$ and [C/Y]-(VT)$_5^{\Psi\ 11,15}$ are the ones with the highest molecular weight ~32 kg/mol (Figure S13 and Table 2).

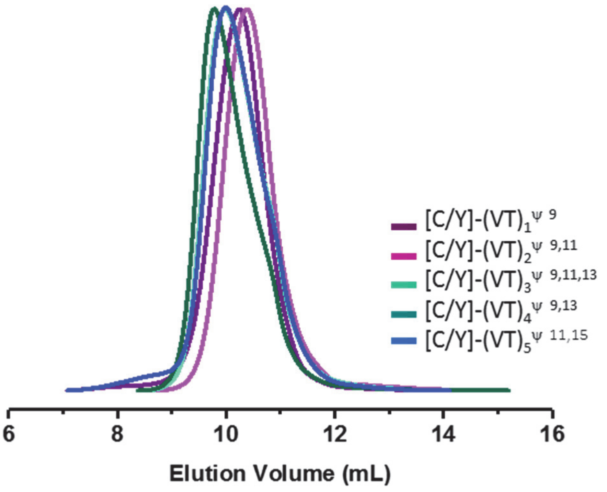


**Figure S13**. GPC measurement of switch-block polymers.

**Table S2**. GPC data

| Polymer | $Mp_{app}$ (g/mol) | Đ | $DP_{n,app}$ |
|---|---|---|---|
| [C/Y]-(VT)$_1^{\Psi\ 9}$ | 31000 | 1.53 | 35 |
| [C/Y]-(VT)$_2^{\Psi\ 9,11}$ | 26000 | 1.46 | 24 |
| [C/Y]-(VT)$_3^{\Psi\ 9,11,13}$ | 21000 | 1.43 | 16 |
| [C/Y]-(VT)$_4^{\Psi\ 9,13}$ | 23000 | 1.41 | 16 |
| [C/Y]-(VT)$_5^{\Psi\ 11,15}$ | 32000 | 1.68 | 19 |

## *5.4 Secondary interactions and switch behavior monitored by circular dichroism*

- **Depsipeptides**

A 250 µM solution of each depsipeptide switch in phosphate buffer 10 mM and at pH 5.5 and pH 7.4 was measured (Figure S14). The measurements of the depsipeptides in acidic conditions show the absence of β-sheet formation (Figure S14a). However, after the adjustment of the pH to 7.4, the O→N-acyl transfer rearrangement took place in the switch segments of the monomer constructs, which restores the native peptide backbone and the (VT)$_n$ domains regain their β-sheet formation tendency. This evidence is showed by the presence of the typical Cotton bands for β-sheets at -214 nm and +193 nm in the CD spectra (Figure S14b). Despite having (VT)$_n$ domains into the sequence, the formation of β-sheets is facilitated as the length of the (VT)$_n$ segments increases, showing more pronounced β-sheet formation in the CD spectra for the longer constructs.

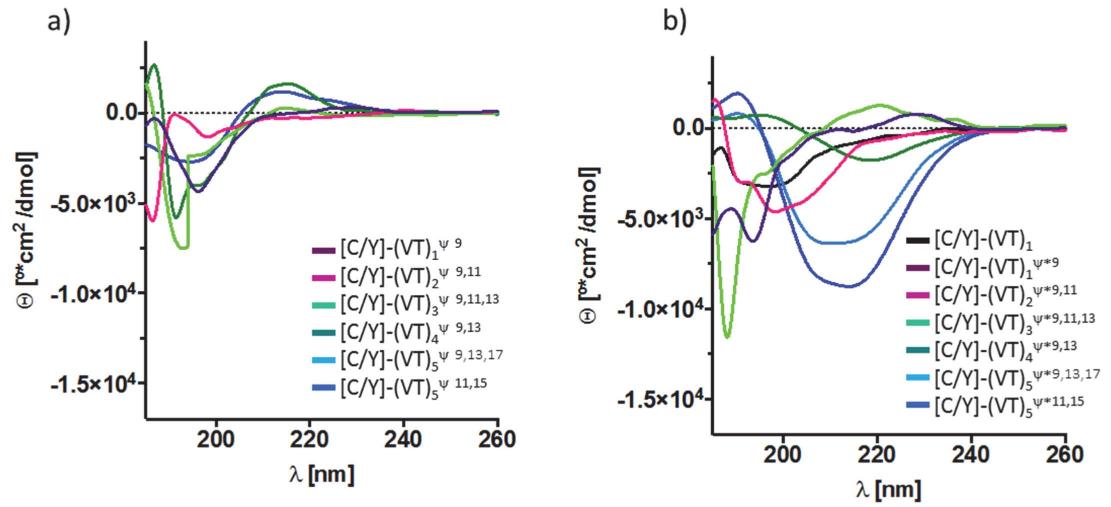


**Figure S14**. CD measurement of depsipeptide switches at a) pH 5.5, and b) pH 7.4 after 40 min.

- **Polymers**

A 0.75 µM solution of each depsipeptide was polymerized according to protocol given in the section 3.4 using an *Ab*PPO4 tyrosinase concentration of 50 U/mL in 1.5 mL. After 30 minutes, 5 µL of 6 M HCl were added (resulting in pH 2 of the sample solution). Afterwards samples were frozen in liquid nitrogen and lyophilized.

- *Polymers at pH 5.5*

The dry polymers where dissolved in phosphate buffer (10 mM) at pH 5.5 to a final concentration of 250 µM and measured after 40 min. (Figure S15a). After 24 h no changes in the CD spectra were observed.

- *Polymers at pH 7.4*

A concentration of 250 µM of polymers in phosphate buffer (10 mM) at pH 7.4 where measured after 20 and 40 min (Figure S15b and Figure S16).

As in the depsipeptide measurements, at pH 5.5 no evidence of β-sheet formation was found. However, at pH 7.4 as the length of the $(VT)_n$ segments in the polymerized depsipeptides increases, more pronounced β-sheet formation is evident in the CD spectra (Figure S15). Moreover, the β-sheet signal observed in the polymers measurements has a more pronounced intensity than its corresponding monomer, showing the presence of a higher β-sheet content in the system (Figure S16).

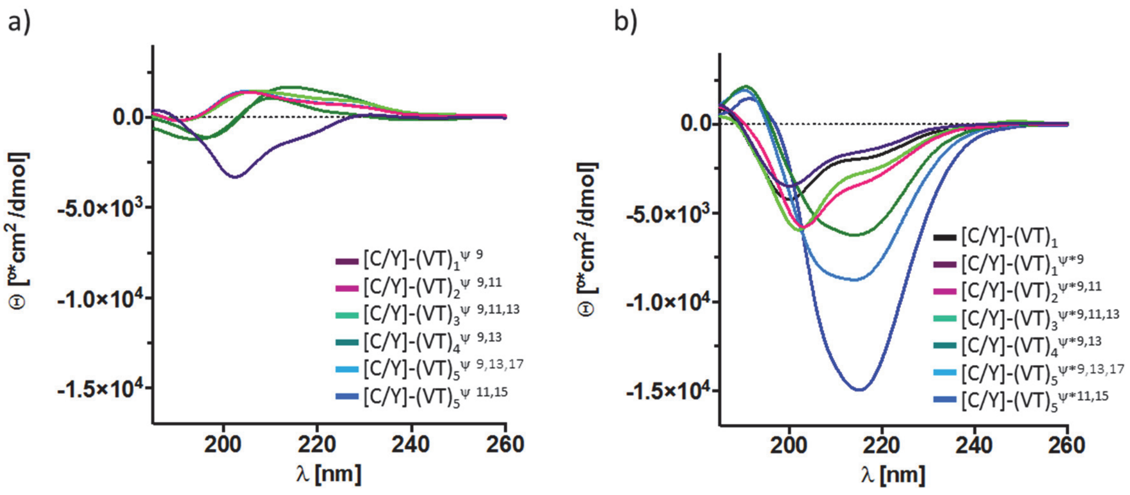


**Figure S15**. CD measurement of polymers at 250 µM in phosphate buffer 10 mM at a) pH= 5.5, and b) pH 7.4 after 40 min.

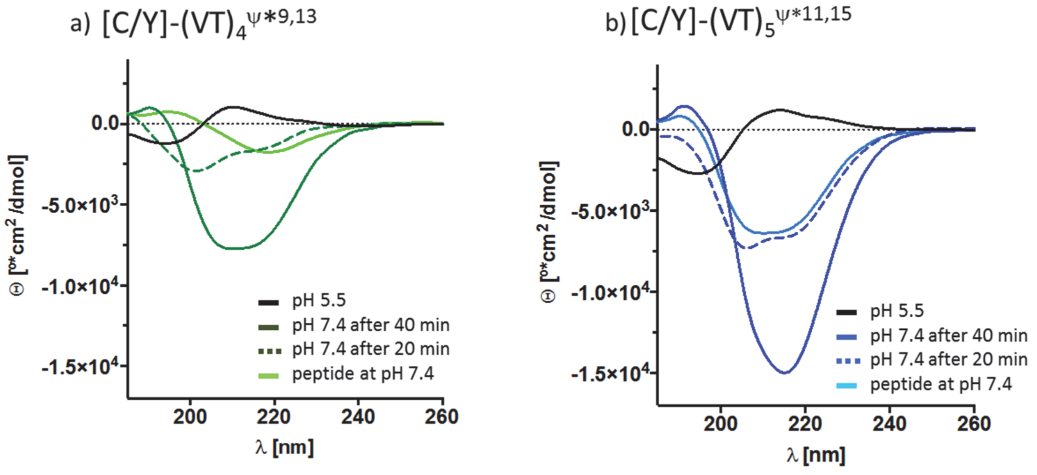


**Figure S16**. CD measurement of depsipeptide and polymer at 250 µM in phosphate buffer 10 mM a) [C/Y]-(VT)$_4$$^{\Psi*9,13}$ and b) [C/Y]-(VT)$_5$$^{\Psi*11,15}$.

## *5.5 Microscopy studies*

Microscopy analysis was performed in order to visualize nanostructures generated by the formation of β-sheet in the switched polymers. Sample concentration was 0.75 µM. Solutions of [C/Y]-(VT)$_4$$^{\Psi\ 9,13}$ and [C/Y]-(VT)$_5$$^{\Psi\ 11,15}$ were polymerized according to the protocol given in the section 3.4 using an *Ab*PPO4 concentration of 50 U/mL in 1.5 mL. After 30 minutes of polymerization, 5 µL of 6 M HCl were added (resulting in pH 2 of the sample solution). Afterwards, samples were frozen in liquid nitrogen and lyophilized. Subsequently, the samples were dissolved in a buffer solution at pH 5.5 and pH 7.4 with a concentration of 0.5 µM for TEM and 0.05 µM for AFM.

- **Transmission electron microscopy (TEM)**

TEM analyses were prepared by drop-casting using 10 µL of the corresponding samples solutions onto a copper grid. The analyses show the absence of β-sheet formation when the polymer is at pH 5.5, while well-defined fibrillar aggregates are observed for switched polymers (Figure S17).

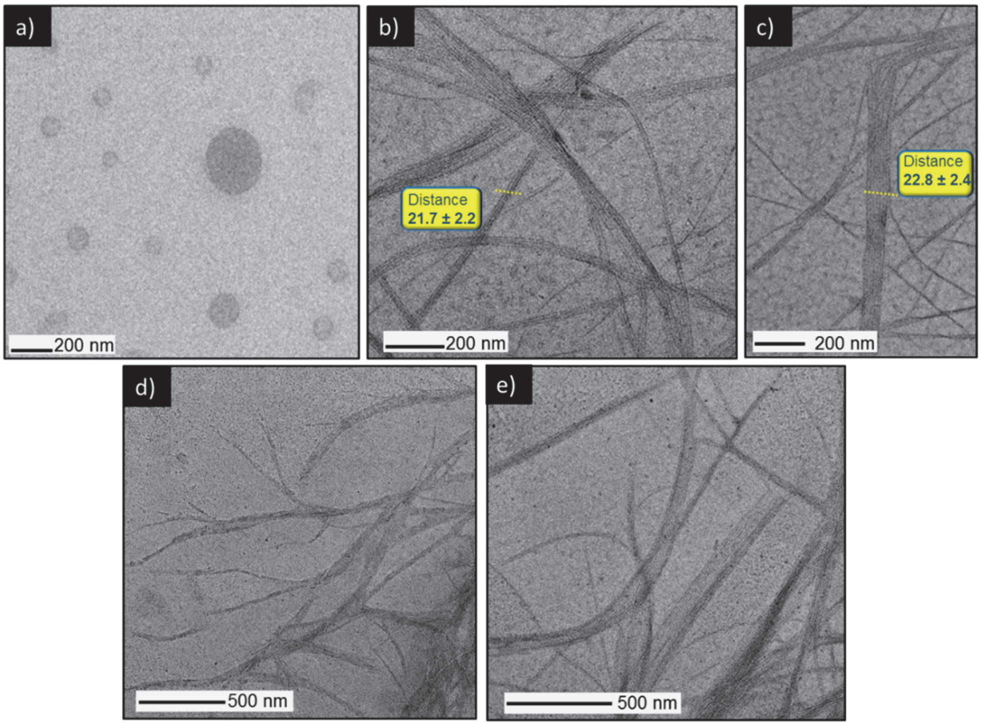


**Figure S17.** TEM images of a) poly([C/Y]-(VT)$_5$$^{\Psi 11,15}$) at pH 5.5, b-c) poly([C/Y]-(VT)$_5$$^{\Psi*9,13}$) at pH 7.4 and d-e) poly([C/Y]-(VT)$_4$$^{\Psi*9,13}$) at pH 7.4.

- **Atomic force microscopy (AFM)**

AFM analysis were prepared by spin coating using 10 µL of a 0.05 µM solution of poly([C/Y]-(VT)$_5$$^{\Psi*11,15}$) at pH 7.4 onto freshly cleaved Mica substrates at 100 rpm followed by 2500 rpm to dry the substrate. In this case, also a well-defined fibrillar aggregates occurs for switched polymers confirming the disturbing effects of the depsi-structure defects (Figure S18).

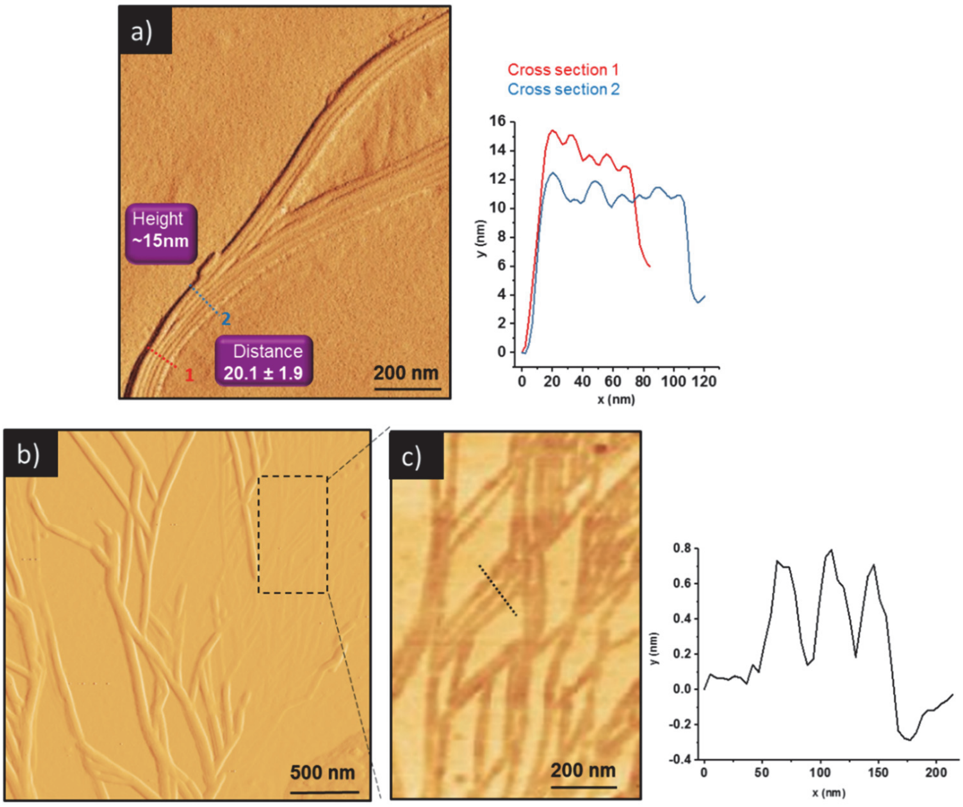


**Figure S18.** AFM images of poly([C/Y]-(VT)$_5$$^{\Psi11,15}$) at pH 7.4 showing the presence of fibers due to the β-sheet formation.

## 5.6 QCM-D experiments on aluminum oxide surface

For QCM measurements sample concentration (0.75 µmol/mL) was reduced and samples were diluted after reaction prior to measurement with degassed Milli-Q water 1:21 v/v (0.03 µmol/mL). Therefore, the citrate buffer with the appropriate pH for the specific experiment (pH 5.5 or pH 7.4, 17 mM) was diluted to 0.8 mM as well for use in equilibration and rinsing steps. All measurements were performed at 100 µL/min flow according to protocol 3.5 on an aluminium oxide coated sensor (QSX 309, Biolin Scientific, Sweden). Changes in frequency (∆f) and energy dissipation (∆D) were recorded for overtones n = 3, 5, 7, 9, 11 and 13. Calculations of adsorbed masses for the polymer coatings were performed with QTools Software (version 3.0.10.286, Biolin Scientific AB, Sweden) by approximation of the recorded data according to the Voight-based model.[10]

### 5.6.1 peptide([C/Y]-(VT)$_5^{\Psi\ 11,15}$) control

As a control experiment QCM-D measurement of the pure depsipeptide was carried out. Therefore, a solution of [C/Y]-(VT)$_5^{\Psi\ 11,15}$ in buffer 17 mM at pH 5.5 (0.75 µmol/mL) was prepared, the sample of 1.5 mL was diluted to 33 mL with degassed Milli-Q water and the sensor was incubated for 5 h followed by buffer rinsing. The observed adsorption is rather weak, but Δf remained constant during buffer rinsing (Figure S19), indicating no washing-off of the formed coating.

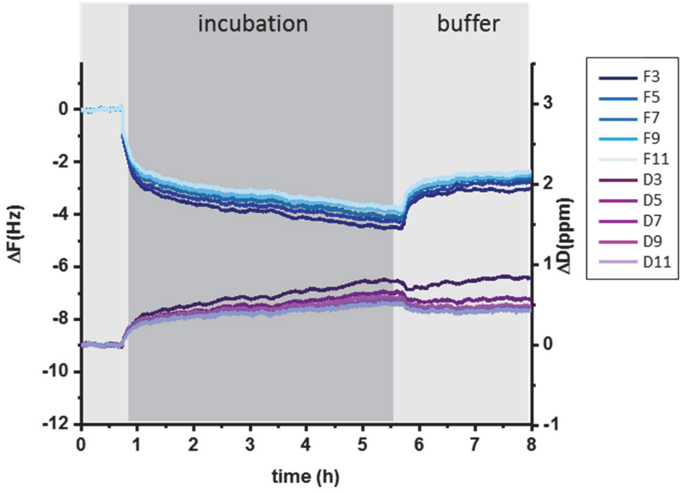


**Figure S19:** QCM-D adsorption and desorption kinetics of peptide([C/Y]-(VT)$_5^{\Psi\ 11,15}$) at pH 5.5 onto surface modification on $Al_2O_3$.

### 5.6.2 poly([C/Y]-(VT)$_5^{\Psi\,11,15}$) and poly([C/Y]-(VT)$_5^{\Psi*11,15}$) coating

[C/Y]-(VT)$_5^{\Psi\,11,15}$ was polymerized for 1 h at pH 5.5 according to the protocol given in the section 3.4 with 0.75 µmol/mL substrate concentration and an *Ab*PPO4 concentration of 50 U/mL.

- ***Coating at pH 5.5***

The sample of 1.5 mL was diluted to 33 mL with degassed Milli-Q water and the sensor was incubated for 4 h followed by buffer rinsing at pH 5.5. The observed adsorption is rather weak, but Δf remained constant during buffer rinsing (Figure S20a, b), indicating again no washing-off of the formed coating.

- ***Coating at pH 7.4***

Afterwards samples were frozen in liquid nitrogen and lyophilized. The polymer was dissolved in 1.5 mL of buffer at pH=7.4 resulting in poly([C/Y]-(VT)$_5^{\Psi*11,15}$). After 40 min, the sample was diluted to 33 mL with degassed Milli-Q water and the sensor was incubated for 4 h followed by buffer rinsing at pH 7.4. The resulting poly([C/Y]-(VT)$_5^{\Psi*11,15}$) showed strong adsorption towards the aluminum oxide surface and during buffer, rinsing Δf remained constant, proving the stability of the polymer coating (Figure S20a, c). Multilayer formation occurs and prevents to reach equilibrium, which is often observed for protein adsorption processes.[11]

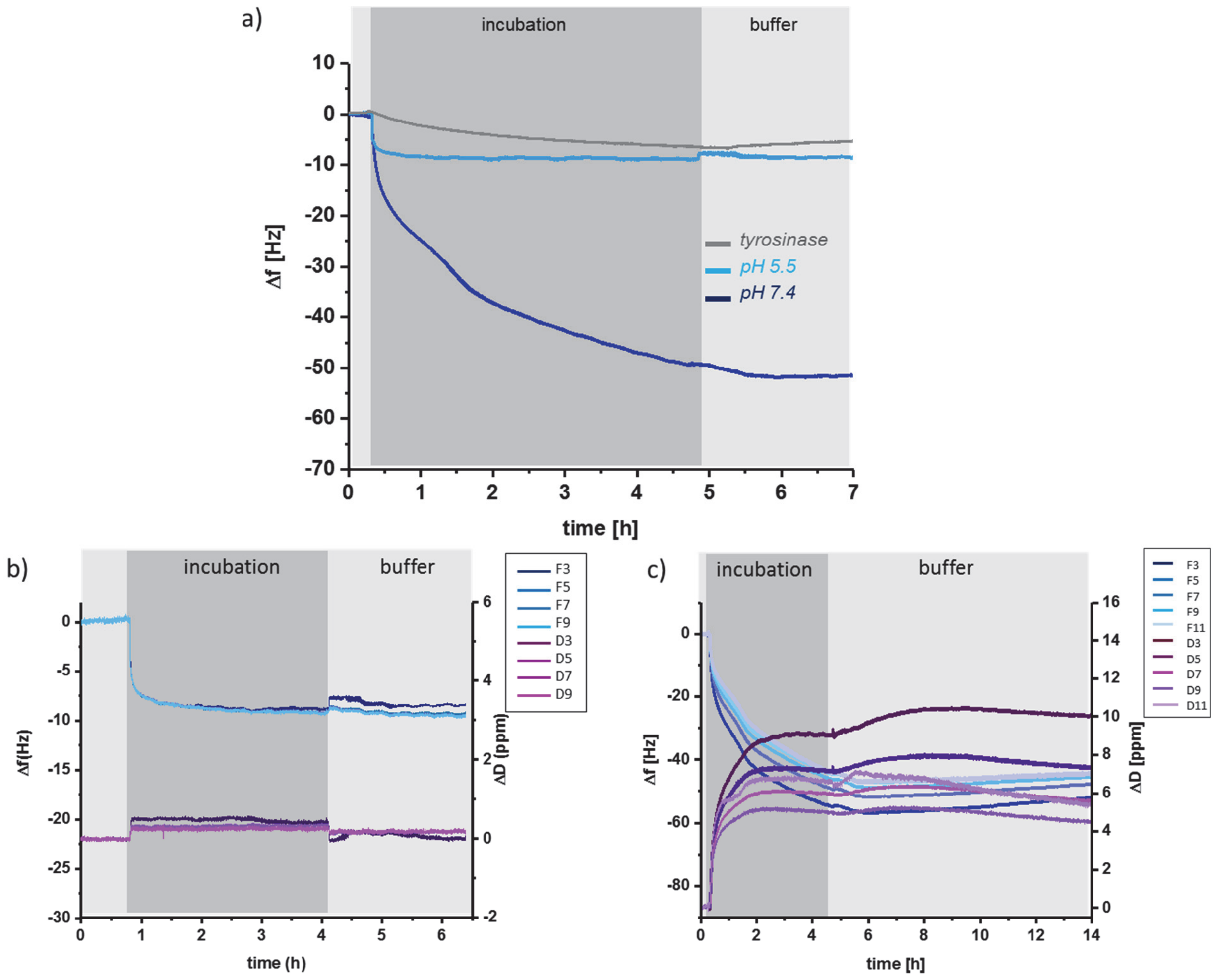


**Figure S20:** a) Comparison of QCM-D adsorption and desorption kinetics of mfp analogues at different pH and enzyme reference on $Al_2O_3$ coated sensors. QCM-D adsorption and desorption kinetics of a) poly([C/Y]-(VT)$_5^{\Psi\,11,15}$) at pH 5.5 and b) poly([C/Y]-(VT)$_5^{\Psi\,11,15}$) at pH 7.4 onto surface modification on $Al_2O_3$.

- ***Rinsing of polymer coating***

To test for coating stability, the poly([C/Y]-$(VT)_5^{\Psi* 11,15}$) coated sensor was rinsed with 599 mM NaCl solution and 4.2 M hypersaline solution for 1 h. The used hypersaline solution was modeled after salt concentrations of Dead Sea[12] water and contained $MgCl_2 \cdot 6H_2O$ (368.0 g/L, 1.81 mol/L), NaCl (97.0 g/L, 1.66 mol/L), $CaCl_2 \cdot 2H_2O$ (63.2 g/L, 0.43 mol/L), KCl (14.9 g/L, 0.20), NaBr (6.82 g/L, 66.33 mmol/L), $Na_2SO_4$ (664.7 mg/L, 4.68 mmol/L) and $NaHCO_3$ (275.6 mg/L, 3.28 mmol/L). The calculated differences in adsorbed masses amounts to 727 ng/cm² (2.4%) for NaCl rinsing and 673 ng/cm² (7.2%) for hypersaline rinsing compared to the initial coating, which demonstrates the coating is highly stable against salinity (Figure S21).

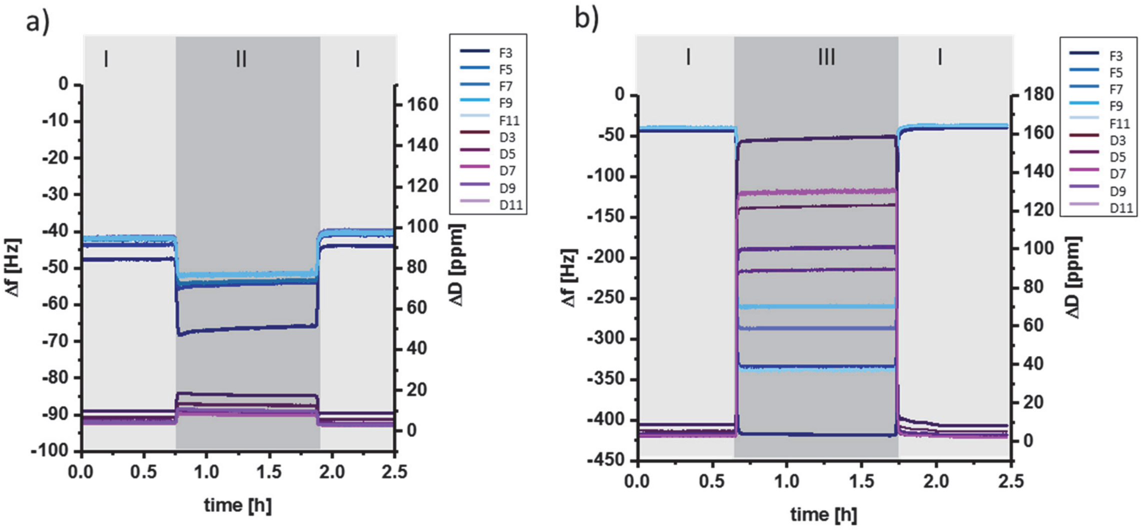


**Figure S21**. QCM-D adsorption and desorption kinetics of rinsing poly([C/Y]-$(VT)_5^{\Psi* 11,15}$) modified $Al_2O_3$ surface at pH 7.4 with a) 599 mM NaCl solution and b) 4.2 M hypersaline solution.

I. citrate buffer solution (0.8 mM, pH 7.4)

II. NaCl solution (599 mM)

III. hypersaline solution (4.2 M)

### 5.6.3 poly([C/Y]-(VT)$_4^{\Psi* 9,11}$) and poly([C/Y]-(VT)$_4^{\Psi* 9,11}$) coating

[C/Y]-(VT)$_4^{\Psi 9,11}$ was polymerized for 1 h at pH 5.5 according to the protocol given in the section 3.4 with 0.75 µmol/mL substrate concentration and an *Ab*PPO4 concentration of 50 U/mL.

- ***Coating at pH 5.5***

The sample of 1.5 mL was diluted to 33 mL with degassed Milli-Q water and the sensor was incubated for 4 h followed by buffer rinsing at pH 5.5. The observed adsorption is rather weak, but Δf remained constant during buffer rinsing (Figure S22a), the formed coating was not washed-off.

- ***Coating at pH 7.4***

The poly([C/Y]-(VT)$_4^{\Psi 9,11}$) was frozen in liquid nitrogen and lyophilized. Later, it was dissolved in 1.5 mL of a pH 7.4 buffer solution to get the poly([C/Y]-(VT)$_4^{\Psi* 9,11}$) and after 40 min it was diluted to 33 mL with degassed Milli-Q water and the sensor was incubated for 4 h followed by buffer rinsing at pH 7.4. The observed adsorption is four times stronger than in the previous case and after buffer rinsing (pH 7.4) Δf remained constant, proving the stability of the polymer coating (Figure S22b).

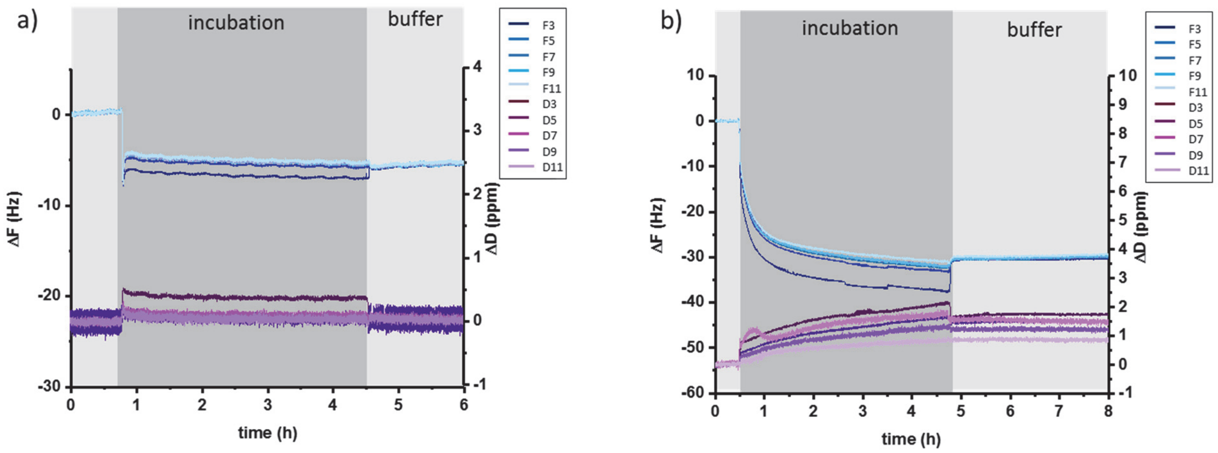


**Figure S22:** QCM-D adsorption and desorption kinetics of a) poly([C/Y]-(VT)$_4^{\Psi* 9,11}$) at pH 5.5 and b) poly([C/Y]-(VT)$_4^{\Psi* 9,1}$) at pH 7.4 on $Al_2O_3$.

- ***Rinsing of polymer coating***

Coating stability of the poly([C/Y]-(VT)$_4^{\Psi* 9,13}$) coated sensor was tested by rinsing with 599 mM NaCl and with hypersaline solution (4.2 M) for 1 h. The calculated difference in adsorbed masses amounts to 538 ng/cm² (10%) for NaCl and to 499 ng/cm² (11%) for hypersaline solution compared to the initial coating, which demonstrates high resistance to salinity (Figure S23).

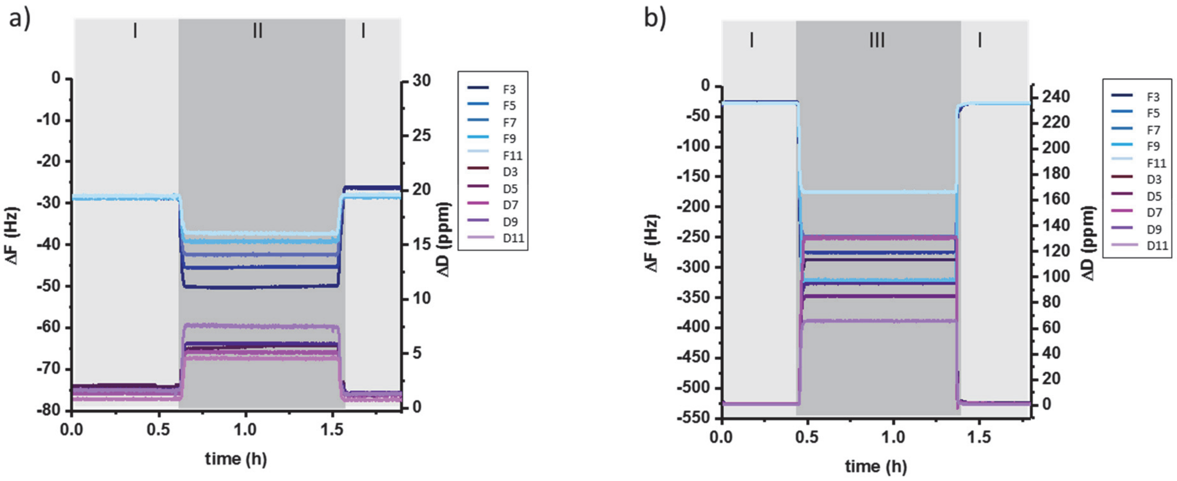


**Figure S23**. QCM-D adsorption and desorption kinetics of rinsing poly([C/Y]-(VT)$_4^{\Psi* 9,13}$)-modified $Al_2O_3$ surface at pH 7.4 with a) 599 mM NaCl solution and b) 4.2 M hypersaline solution.

I. citrate buffer solution (0.8 mM, pH 7.4)

II. NaCl solution (599 mM)

III. hypersaline solution (4.2 M)

## 5.7 Soft colloidal probe atomic force microscopy to characterize adhesion properties

- *Adhesion properties of freshly prepared coatings*

For adhesion measurements, clean, plasma activated glass slides were coated with the polymer [C/Y]-(VT)$_5$$^{\psi}$ [11,15] at pH 5.5 and pH 6.8 for one hour. The glass slides were cleaned prior to use in an ultrasonic bath first in acetone, than in 2-propanol, ethanol, ethanol-water mixture 50:50, and pure milliQ water for 10 min followed by excessive rinsing with MilliQ-water after each step. Therefore, coating solutions were prepared according to procedure 3.4 using a unimer concentration of 0.75 mM and an *Ab*PPO4 concentration of 50 U/mL. Freeze dried samples were dissolved in 200 µL of the corresponding pH buffer solution and put on glass slides. The glass slides were incubated with this solution for one hour.

After the polymerization, the substrates were cleaned with buffer solution and used directly for the AFM experiments. The samples were transferred to a closed fluid cell and fixed to the fluid cell at the edges with two screws. During the measurements, the cell was completely filled with the corresponding buffer.

On each sample, adhesion forces were measured on at least three different positions on the sample, by taking 20 x 20 µm, 4 points force maps at at least three spots on each sample. To generate a high contact area, load forces of 100 and 500 nN were used. The time in contact was 10 sec. During adhesion measurements, the surface of clean, plasma-activated glass at the corresponding buffer was used as a reference.

To gain the adhesive data, the raw data set was transformed to force versus deformation curves by taking the spring constant, lever sensitivity, and the lever deflection in contact into account.[3, 13] From these curves, the adhesion force $F_A$ (maximum negative force during retraction out of contact) is calculated and converted to the work of adhesion per unit area $W_{adh}$ according to the Johnson-Kendall-Roberts (JKR) model. [14]

$$W_{adh} = \frac{2F_A}{3\pi R_{eff}} \qquad \text{(eq. S2)}$$

$\frac{1}{R_{eff}} = \frac{1}{R_1} + \frac{1}{R_2}$ where $R_{eff}$ is the effective radius of the probe and the substrate, $R_1$ is the radius of the probe and $R_2$ is the radius of the substrate. Because of the geometry (sphere-plane) the radius of the substrate is infinite, hence $R_{eff} = R_1$.

A smooth and stable polymer layer was observed at pH 5.5. The adhesion values increase with the load force from 100 nN to 500 nN. The corresponding work of adhesion is $W_{adh\text{-pH5.5}}$=0.23±0.07 mJ/m$^2$ for 100 nN and $W_{adh\text{-pH5.5}}$=0.54±0.09 mJ/m$^2$ for 500 nN, respectively. As demonstrated in Figure S24a and b the adhesion values of poly([C/Y]-(VT)$_5^{\Psi*11,15}$) at pH 6.8 drop after several measurements with soft colloidal probe and then remain constant.

To determine the adhesion values, a huge contact area between the thin polymer layer and the PDMS probe is necessary. Due to the non-covalent bonding of the polymer layer to the substrate, it is possible that some polymer is collected by the probe with increasing number of contacts, which may cause change of adhesion values after several contacts. This behavior is demonstrated in Figure S24c, dark blue line represents the first measurements at pH 6.8 while the dotted dark blue line shows the retraction of the soft colloidal probe after several contacts. We assume that some polymer material sticks to the PDMS bead and a combination of cohesion and adhesion is measured. Nevertheless, the calculated values are at least two times higher for pH 6.8 in comparison to the lower pH.

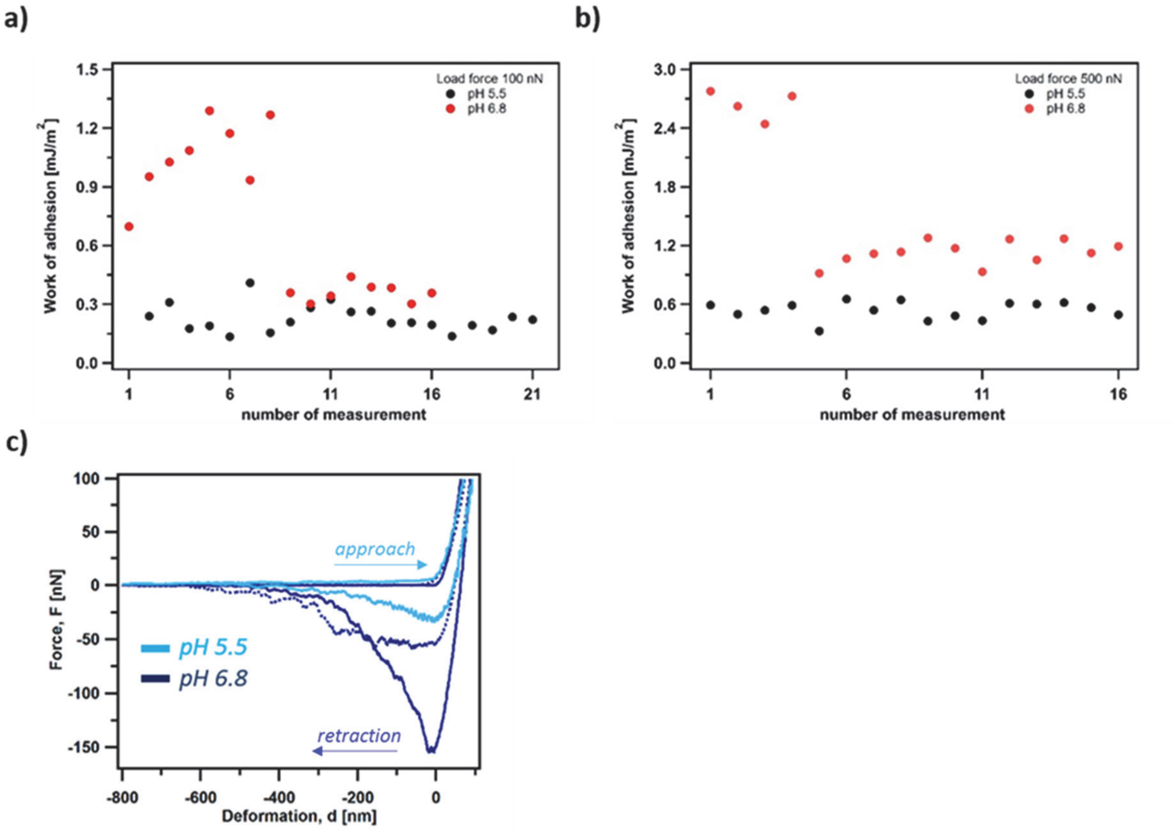


**Figure S24**. Characterization of adhesion on mussel inspired polymer by soft colloidal probe AFM. Calculated work of adhesion per unit area by JKR model for all measurements at pH 5.5 and pH 6.8 at loading forces of a) 100 nN and b) 500 nN c) Representative force vs. deformation curves for pH 5.5 and pH 6.8. at 500 nN loading force. Dark blue dotted line shows the adhesion forces of soft colloidal probe with some collected polymer material, after several measurements.

- *Switchable adhesion in situ experiments*

For *in situ* adhesion measurements a glass cover was coated with the poly([C/Y]-(VT)$_5$ $^{\Psi\ 11,15}$) at pH 5.5 for 2 h of coating time with the same procedure as described above. Two 4 points force maps were done on the surface at different positions. After each force map, the cantilever was rinsed with an excessive amount of water to prevent contamination of the cantilever with the polymer chains in order to access the value of adhesion itself. After rinsing, force curves were recorded on the cleaned glass at the buffer solution. The corresponding work of adhesion on the polymer layer measured at pH 5.5 is $W_{adh\text{-}pH5.5}$=0.60±0.19 mJ/m$^2$ for 500 nN (Figure S 25a). To demonstrate that the switch in the polymer backbone takes place due to a pH change from pH 5.5 to pH 6.8, 12 mL of pH 6.8 buffer solution was pumped through the cell using microfluidic pump with the flow rate of 3 mL/min. The reference was recorded at the pH 6.8 buffer solution to ensure the reliability of the measurements. After an equilibration time of approx. 40 minutes at pH 6.8, 4 force maps were recorded on the surface at different positions, following the same procedure used for pH 5.5. Considering the same number of points (n=8), the value of adhesion force after pH changing, increased dramatically showing a work of adhesion measured at pH 6.8 of $W_{adh\text{-}pH6.8}$=1.80±0.25mJ/m$^2$ for 500 nN. The results emphasize that the change in adhesion in the Figure S25 is due to a switching of the polymer. As evident in previous measurements, the probe collects some adhesive polymer in the duration of the repetitive measurements, causing higher noise and scattering of adhesion values with increased number of contacts. If the probe surface is partially modified by adhesive polymers throughout the measurements of several force curves, a mixture between cohesion and adhesion was measured that is not straightforward to interpret. Calculation of adhesion forces considering the stable values from the first half of the measurement cycles (n=8 of 16) avoids misinterpretation of adhesion values. However, the average work of adhesion considering the complete set of measurements at pH 6.8 that includes the pick-up bias values (n=16 of 16) provides not significantly reduced values of $W_{adh\text{-}pH6.8}$=1.54±0.37mJ/m$^2$ for 500 nN. It is important to mention that during all experiments no significant change in the adhesion values and force curves shape for the reference was found.

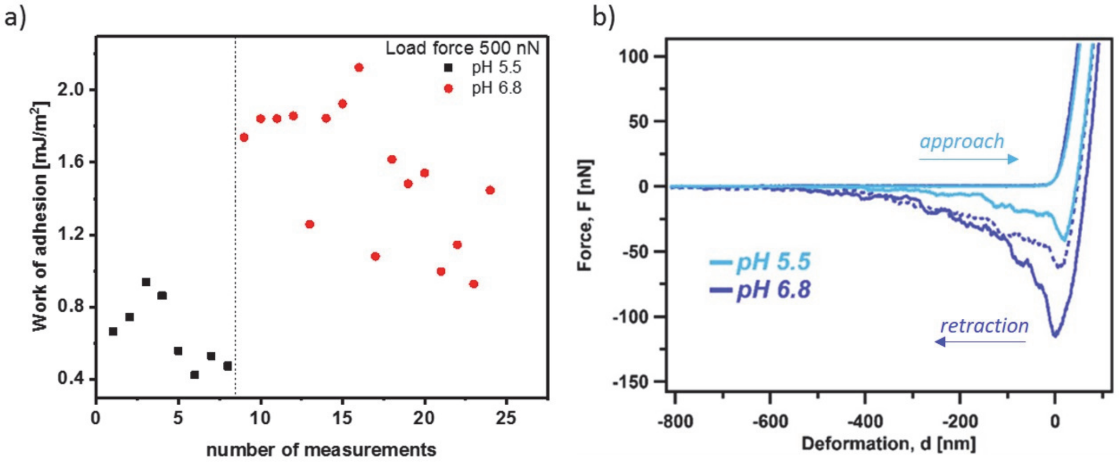


**Figure S25**. *in situ* characterization of the adhesion behavior on poly([C/Y]-(VT)$_5$ $^{\Psi\ 11,15}$) by soft CP-AFM. Calculated work of adhesion per unit area by JKR model for all measurements at pH 5.5 and pH 6.8 at loading forces of a) 500 nN. b) Representative force vs. deformation curves for pH 5.5 and after pH change to pH 6.8 at 500 nN loading force.

## 5.8 Depth-sensing nanoindentation measurements

Two different 0.75 µM solutions of the depsipeptide [C/Y]-(VT)$_5^{\Psi 11,15}$ were polymerized according to the protocol given in the section 3.4 using water at pH 5.5 in stead of buffer solution to avoid the formation of salts on the films. After 45 minutes, 1.3 µL of 6 M HCl were added (resulting in pH 2 of the sample solution). Afterwards samples were frozen in liquid nitrogen and lyophilized.

- *Film preparation for poly([C/Y]-(VT)$_5^{\Psi 11,15}$) at pH 5.5*

One of the sample was dissolved in a water solution at pH 5.5 (0.75 µmol/mL) and 10 µL of the solution were deposited on the silicon wafer surface by drop casting.

- *Film preparation for poly([C/Y]-(VT)$_5^{\Psi *11,15}$) at pH 6.8*

The other sample was dissolved in a water solution at pH 6.8 (0.75 µmol/mL). After 3h, 10 µL of the solution were deposited on the silicon wafer surface by the drop casting method.

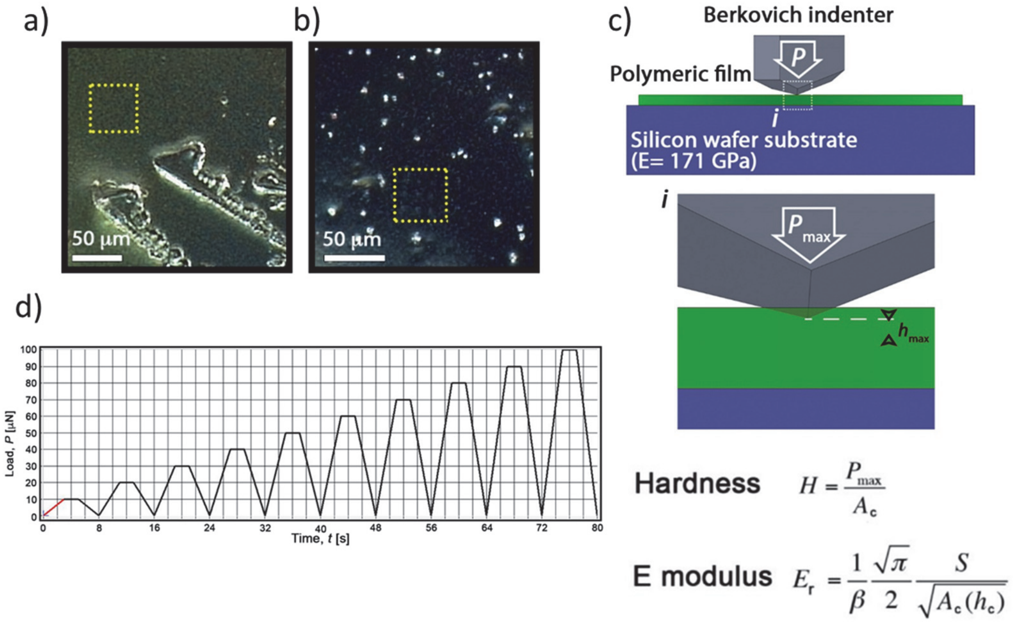


**Figure S26**. Illustration of the probed areas on films of (a) poly([C/Y]-(VT)$_5^{\Psi 11,15}$) at pH 5.5 and (b) poly([C/Y]-(VT)$_5^{\Psi *11,15}$) at pH 6.8. c) Graphic concept of depth-sensing nanoindentation. The films formed on a silicon wafer substrate were probed using a Berkovich tip and (d) a depth profiling cyclic load function.

## References


[1] M. Pretzler, A. Bijelic, A. Rompel, *Sci. Rep.* **2017**, *7*, 1810-1819.
[2] J. L. Hutter, J. Bechhoefer, *Review of Scientific Instruments* **1993**, *64*, 1868-1873.
[3] H.-J. Butt, B. Cappella, M. Kappl, *Surface Science Reports* **2005**, *59*, 1-152.
[4] W. C. Oliver, G. M. Pharr, *J. Mater. Res.* **1992**, *7*, 1564-1583.
[5] J. B. W. Hammond, R. Nichols, *J. Gen. Microbiol.* **1976**, *93*, 309.
[6] D. S. Hwang, H. Zeng, A. Srivastava, D. V. Krogstad, M. Tirrell, J. N. Israelachvili, J. H. Waite, *Soft Matter* **2010**, *6*, 3232-3236.
[7] F. W. Studier, *Protein Expr. Purif.* **2005**, *41*, 207-234.
[8] H. W. C. Duckworth, J. E. , *J. Biol. Chem.* **1970**, *245*.
[9] J. Yang, V. Saggiomo, A. H. Velders, M. A. Cohen Stuart, M. Kamperman, *PLoS One* **2016**, *11*, e0166490.
[10] a) H. L. Bandey, A. Robert Hillman, M. J. Brown, S. J. Martin, *Faraday Discussions* **1997**, *107*, 105; b) M. V. Voinova, M. Rodahl, M. Jonson, B. Kasemo, *Phys. Scr.* **1999**, *59*, 391-396.
[11] S. M. Notley, M. Eriksson, L. Wagberg, *J. Colloid Interface Sci.* **2005**, *292*, 29-37.
[12] I. Steinhorn, G. Aaaf, J. R. Gat, A. Nishry, A. Nissenbaum, M. Stiller, M. Beyth, D. Neev, R. Garber, G. M. Freidman, W. Weiss, *Science* **1979**, *206*, 55.
[13] M. Seuss, F. A., **2018**.
[14] L. Johnson, K. Kendall, A. D. Roberts, *Roy. Soc. A.-Math. Phy.* **1971**, *324*, 301-313.
